# Small RNAs of *Haloferax mediterranei*: Identification and Potential Involvement in Nitrogen Metabolism

**DOI:** 10.3390/genes9020083

**Published:** 2018-02-10

**Authors:** Gloria Payá, Vanesa Bautista, Mónica Camacho, Natalia Castejón-Fernández, Luís A. Alcaraz, María-José Bonete, Julia Esclapez

**Affiliations:** 1 Agrochemistry and Biochemistry Department, Biochemistry and Molecular Biology Division, Faculty of Science, University of Alicante, Ap 99, E-03080 Alicante, Spain; gloria.paya@gmail.com (G.P.); vanesa.bautista@ua.es (V.B.); camacho@ua.es (M.C.); luis.alcaraz@ua.es (L.A.A.); 2 Bioarray, S.L., 03202 Alicante, Spain; natalia.castejon@bioarray.net

**Keywords:** haloarchaea, sRNA, nitrogen assimilation, RNA-Seq

## Abstract

Small RNAs have been studied in detail in domains Bacteria and Eukarya but, in the case of the domain Archaea, the knowledge is scarce and the physiological function of these small RNAs (sRNAs) is still uncertain. To extend the knowledge of sRNAs in the domain Archaea and their possible role in the regulation of the nitrogen assimilation metabolism in haloarchaea, *Haloferax mediterranei* has been used as a model microorganism. The bioinformatic approach has allowed for the prediction of 295 putative sRNAs genes in the genome of *H. mediterranei*, 88 of which have been verified by means of RNA-Sequencing (RNA-Seq). The secondary structure of these sRNAs and their possible targets have been identified. Curiously, some of them present as possible target genes relating to nitrogen assimilation, such as glutamate dehydrogenase and the nitrogen regulatory PII protein. Analysis of RNA-Seq data has also revealed differences in the expression pattern of 16 sRNAs according to the nitrogen source. Consequently, RNomic and bioinformatic approaches used in this work have allowed for the identification of new sRNAs in *H. mediterranei*, some of which show different expression patterns depending on the nitrogen source. This suggests that these sRNAs could be involved in the regulation of nitrogen assimilation and can constitute an important gene regulatory network.

## 1. Introduction

Small RNAs (sRNAs) play an essential role in the post-transcriptional regulation of many cellular processes in all domains of life, i.e., Eukarya, Bacteria, and Archaea. In eukaryotes, there are different classes of these, the best studied being microRNA (miRNAs), small interference RNA (siRNAs), and piwi-interacting RNAs (piRNAs). These are approximately 20–30 nucleotides (nt) in length and are involved in development, cellular activities, and different physiology processes [1,2,3]. In Archaea and Bacteria, sRNAs are much longer than eukaryotic small non-coding RNAs, which range in length from 50 to 500 nt [4,5,6,7]. Different mechanisms of action of sRNAs have been described, most of which affect the translation of the target messenger RNA and/or its stability [4]. Hence, they seem to be involved in the post-transcriptional regulation of metabolism, stress response, virulence processes, and so on. Depending on the location of their targets, sRNAs are classified into two groups: *trans*-encoded and *cis*-encoded sRNAs [8]. *Trans*-encoded sRNAs are those that are encoded within intergenic regions of the genome: they show a stable secondary structure and act on target sequences located at different positions in the genome. The complementarity between the sRNA and its target sequence is not complete and, for this reason, they require the presence of RNA chaperones to facilitate nucleotide binding [9]. In contrast, *cis*-encoded sRNAs originate in the nonsense strand of an open reading frame (ORF). Generally, that ORF corresponds to the sRNA target which presents complete complementarity to the *cis*-encoded sRNA. In addition, there have also been other types of RNA identified in Archaea: small nucleolar RNAs (snoRNAs) involved in the modifications of ribosomal RNA, whose presence was originally believed to be restricted to eukaryotic organisms [10]; sRNAs involved in the clustered regularly interspaced short palindromic repeats (CRISPR)/Cas prokaryotes immune system called crRNAs [11]; and sRNAs that derive from transfer RNAs called tRFs [12].

In Eukarya and Bacteria, many sRNAs have been characterised in detail. However, in domain Archaea, though there has been considerable progress in recent years, the number of sRNAs characterised is considerably lower than those in the other two domains [5,13,14]. The application of bioinformatic approaches and high-throughput sequencing systems for the analysis of complementary DNA (cDNA) libraries, RNA-Sequencing (RNA-Seq), have made it easier to understand the transcriptome and discover new sRNAs. However, to date, the identification of sRNAs using RNA-Seq analysis has only been carried out on seven species of Archaea under specific conditions: *Archaeoglobus fulgidus* [15], *Sulfolobus solfataricus* [16], *Pyrococcus abyssi* [10], *Methanosarcina mazei* [17], *Haloferax volcanii* [13], *Pyrobaculum sp.* [18], and *Thermococcus kodakaraensis* [19]. In these studies, it has been shown that the majority of sRNAs identified in Archaea are not conserved, even within species of the same genus [5]. This fact has also been observed in Bacteria, so it appears that the evolution of sRNA genes in prokaryotic organisms is greater than that of genes which encode proteins. Despite the large number of sRNAs identified in Archaea in recent years, very little is known about their biological functions and mechanisms of interaction, with their possible targets still an unknown and unexplored area of research.

*Haloferax mediterranei* is an extremely halophilic archaeon which belongs to the lineage of Euryarchaeota. This microorganism grows optimally at 2.5 M NaCl [20] in a defined medium with glucose as a carbon source and nitrate, nitrite, amino acids, or ammonium as sole nitrogen sources under aerobic conditions [21,22]. Most of the studies focused on nitrogen metabolism in halophilic archaea have been conducted using *H. mediterranei* as a haloarchaeal model. Specifically, biochemical, physiological, and transcriptomic studies of the assimilatory pathway in the presence of different nitrogen sources have been previously performed [22,23,24,25], as well as the development of molecular biology tools in *H. mediterranei* [26]. These results have revealed that the assimilatory pathway is highly regulated at a transcriptional level. However, little is known about the global regulatory networks that allow for the survival of this microorganism under stress or nitrogen starvation conditions. Besides transcriptional regulators analyses, the number of studies which show that sRNA is involved in a great variety of adaptive cellular responses to different stresses have surprisingly increased in the recent years. 

In this work, we present an analysis of putative sRNAs in *H. mediterranei* expressed under two different nitrogen sources, nitrate and ammonium, using a combination of RNA-Seq and bioinformatic approaches. The main aim of this work is to extend the understanding of the global regulation network of the assimilatory pathway in *H. mediterranei*, specifically, and in domain Archaea, generally.

## 2. Materials and Methods

### 2.1. Strains and Growth Conditions

*H. mediterranei* strain R4 (ATCC 33500^T^) was grown at 42 °C with aeration at 225 rpm, contained in a 25% (*w/v*) mixture of inorganic salts (25% salt water) [27]. The pH value was adjusted to 7.3. *H. mediterranei* was grown in two different nitrogen sources, in a defined medium which contained 40 mM KNO_3_ or 40mM NH_4_Cl and supplemented with 5 g/L glucose, 0.0005 g/L FeCl_3_, and 0.5 g/L KH_2_PO_4_. Three independent biological replicates of each condition were employed.

### 2.2. Bioinformatic Prediction of small RNAs

The library of candidate sRNAs was constructed from putative sRNAs obtained in four different species of Archaea: *H. volcanii* [28], *M. mazei* [17], *S. solfataricus* [16], and *A. fulgidus* [15]. All putative sRNA sequences predicted from these four species were compared with the genome of *H. mediterranei* strain R4 (Genbank numbers: CP001868.2, CP001869.1, CP001870.1, and CP001871.1) using BLASTn [25]. The sequences obtained using the candidate sRNAs of other species (*E*-value < 0.05, *p*-value < 0.05) were prioritised using a function of identity, lower number of gaps (maximum 3), and lower number of mismatches.

### 2.3. RNA Isolation

For RNA isolation, *H. mediterranei* was grown in the presence of two different nitrogen sources, nitrate and ammonium, to mid-exponential phase. The mid-exponential growth phase was reached at different times and values of OD600 nm depending on the nitrogen source [23]. RNA was isolated with the *mir*Vana^TM^ miRNA isolation kit (Ambion, Thermo Fisher Scientific, Waltham, MA, USA) following product specifications. Afterwards, the RNA samples were treated with Turbo DNase (Ambion, Thermo Fisher Scientific, Waltham, MA, USA). RNA concentration was analysed by means of a Nanodrop ND-100 Spectrophotometer (Thermo Fisher Scientific, Waltham, MA, USA), and the quality was analysed using the Small RNA Analysis Kit on Agilent 2200 Tapestation (Agilent Technologies, Santa Clara, CA, USA), respectively.

### 2.4. Library of Complementary DNA Preparation and Sequencing

Library preparation and sequencing were performed by the Bioarray, S.L. company (Alicante, Spain). sRNA libraries were constructed using TruSeq Small RNA Library Prep (Illumina, San Diego, CA, USA) and sequenced on an llumina HiSeq 2500 system using a 50-base pair (bp) read length. The corresponding FASTQ files were obtained as a result.

### 2.5. RNA-Sequencing Bioinformatic Analysis

Raw reads from each FASTQ file were aligned into BAM files—compressed binary file used to represent aligned sequences up to 128Mb [29] for each sample of each condition—through bowtie2 2.3.0 [30] using the *H. mediterranei* ATCC 33500 genome (Genbank numbers: GCF_000306765.2_ASM30676v2; Assembly: GCA_000306765.2) as a reference. Seven ammonium BAM files and eight nitrogen BAM files were viewed in the Integrative Genomics Viewer (IGV) program [31,32], alongside the annotated *H. mediterranei* genome.

The raw readings, which aligned at the positions of the library of candidate sRNAs obtained from the different species against the *H. mediterranei* genome, were analysed manually using the Integrative Genomics Viewer (IGV). Only sequences with a score of at least 20 (frequency of reads in high-throughput sequencing) and that were present in the six samples of each condition were selected as sRNAs in *H. mediterranei*. The candidate sRNAs were classified as *cis*-encoded sRNA if they were encoded in the reverse direction of ORF, as *trans*-encoded sRNA if they were in intergenic regions, and as crRNA if they were in a CRISPR array. Since the size of the readings was generally greater than the homology shown in the different species analysed, the BAM files of each condition were pooled, thus, increasing the coverage of the readings, allowing more precise limiting of the sRNAs location in the *H. mediterranei* genome. Once the positions were obtained, the approximate sizes of the sRNAs were calculated. In addition, the genetic environment of each sRNA was analysed manually. 

The counts of the sRNAs of *H. mediterranei* were obtained using the intersection nonempty feature from the HT-Seq program [33]. In this way, for each position *i* in the read, a set *S(i)* is defined as the set of all features overlapping the *i* position. Then, if *S* contains precisely one feature, the read is counted for this feature. If *S* is empty, the read is counted as no feature. Finally, if *S* contains more than one feature, the read is considered as ambiguous and is not counted for any features.

Differential expression analysis in the function of the nitrogen source (nitrate/ammonium) was performed using the DESeq2 library from the Bioconductor 3.5 package [34] obtaining the fold-change of each sRNA (*p*-value < 0.01 and *p*-adj < 0.05). Through this method, the variance-mean dependence in count data was estimated and tested for differential expression; this was based on a model using the negative binomial distribution between nitrate and ammonium group samples.

Both raw (FASTAQ files) and processed data (normalised counts) are available on the Gene Expression Omnibus (GEO) database (Series entry number: GSE108616) [35].

Mfold was used to predict the secondary structure of sRNAs obtained [36]. Potential gene targets for each sRNA were identified using TargetRNA2 [37]. Moreover, the *cis*-encoded sRNAs antisense of characterised ORF were analysed using IntaRNA [38]. BLASTn [25] was used to search homology regions in another organism.

### 2.6. Validation of sRNAs Using Reverse Transcription Polymerase Chain Reaction

The validation of 20 sRNAs was performed by reverse transcription polymerase chain reaction (RT-PCR), prioritising those with differential expression based on the nitrogen source and/or target genes of known functions. The RNA was isolated as described before. Between 0.5 and 0.8 µg of DNA-free RNA was used for the synthesis of cDNA using M-MuLV Reverse Transcriptase (Thermo Fisher Scientific, Waltham, MA, USA) and random hexamer primer (Thermo Fisher Scientific, Waltham, MA, USA) according to the manufacturer’s instructions. Negative controls for reverse transcription polymerase chain reaction (RT-PCR) were prepared by omitting reverse transcriptase and cDNA. The oligonucleotides used to carry out the RT-PCR were designed based on the sequence of sRNA candidates (Table S1). Amplified products were analysed using 3% agarose gel electrophoresis with GeneRuler^TM^ Low Range DNA Ladder (Thermo Fisher Scientific, Waltham, MA, USA) run in parallel. PCR products were purified with the Illustra^TM^ GFX^TM^ PCR DNA and Gel Band Purification Kit (GE Healthcare, Little Chalfont, UK) and confirmed by Sanger sequencing (Stabvida, Caparica, Portugal).

## 3. Results

### 3.1. Identification of Putative small RNAs in Haloferax mediterranei

The library of candidate sRNAs was generated from putative sRNAs obtained in four different species of domain Archaea: *H. volcanii* [28], *M. mazei* [17], *S. solfataricus* [16], and *A. fulgidus* [15]. Since the candidate sRNAs of *H. volcanii* aligned at many positions in the genome of *H. mediterranei* (*E*-value < 0.05, *p*-value < 0.05), the results obtained were prioritised by a function of identity, lower number of gaps (maximum 3), and lower number of mismatches. Given the evolutionary distance, the candidate sRNAs of the remaining species aligned in fewer positions (*E*-value < 1) than *H. volcanii* in the genome of *H. mediterranei*, so it was not necessary to prioritise in the same way. From the bioinformatic analysis, a library of 295 candidate sRNAs in *H. mediterranei* were obtained (Table 1). The number of mismatches allowed depends on the length of the sequence, permitting a greater number of mismatches in larger sequences with a high identity percentage. The results obtained after alignment with the candidate sRNAs of other species are shown in detail in the supplementary material (Tables S2–S5).

### 3.2. Verification of Predicted 295 sRNAs Using RNA-Sequencing

The 295 sRNAs that were bioinformatically predicted were verified with the RNA-Seq results of the *H. mediterranei* ATCC33500. The raw aligned readings obtained from the analysis of RNA-Seq results and the 295 candidate sRNA sequences obtained by means of the bioinformatic approach were manually verified using the high-performance visualisation tool, IGV [31,32]. The transcripts expressed from intergenic regions, antisense to the characterised ORFs, and in the CRISPR array were classified in *trans*-encoded sRNA, *cis*-encoded sRNA, and crRNA, respectively. Due to the size of the readings being generally greater than the homology shown in different species being analysed, the BAM files of each condition were pooled, thus increasing the coverage of the readings and allowing for more accurate localization of the sRNAs in the *H. mediterranei* genome. Once the positions were obtained, the approximate size of the sRNAs was calculated. They were in the range of 20–500 nt. The results of the genetic environment analysis of the 88 verified sRNAs in *H. mediterranei* and their sequences are shown in Table 2 and Table S6, respectively. 

### 3.3. Bioinformatic Analysis of Putative Small RNAs in H. mediterranei

#### 3.3.1. Identification and Classification of small RNAs Verified by RNA-Sequencing

The results of the localisation and classification of the sRNAs verified by RNA-Seq reveals that 58 sRNAs of the 116 sRNAs predicted by homology with *H. volcanii* (Table 1 and Table S2) could be assigned as sRNAs in *H. mediterranei.* There were 56 sRNAs on the chromosome and two on the plasmid pHM500. According to the classification established above, 51 sRNAs are intergenic sRNAs, six are antisense sRNAs, and one is crRNA. The homology analysis performed with *M. mazei* results in 55 predicted sRNAs (Table 1 and Table S3), of which 16 could be sRNAs in *H. mediterranei.* Twelve sRNAs are located on the chromosome, three on the plasmid pHM500, and one on the plasmid pHM100. Related to the classification according to the location of the target, 11 sRNAs are intergenic sRNAs and five antisense. Of the 47 sRNAs predicted by homology with *S. solfataricus* (Table 1 and Table S4), only eight sRNAs were identified in the results of RNA-Seq. These are located on the chromosome, and are classified as four intergenic sRNAs and four antisense sRNAs. From the analysis with *A. fulgidus* (Table 1 and Table S5), only six candidate sRNAs from a total of 74 sRNAs predicted by homology could be assigned as sRNAs. As in the analysis of *S. solfataricus,* all of them are located on the chromosome, with four being intergenic sRNAs and two being antisense sRNAs. From a total of 88 sRNAs verified in *H. mediterranei* by RNA-Seq, 93.18% were located on the chromosome, with most of them (79.54%) being intergenic (*trans*-encoded sRNA).

#### 3.3.2. Structure and Targets of 88 small RNAs

**Figure 1:**
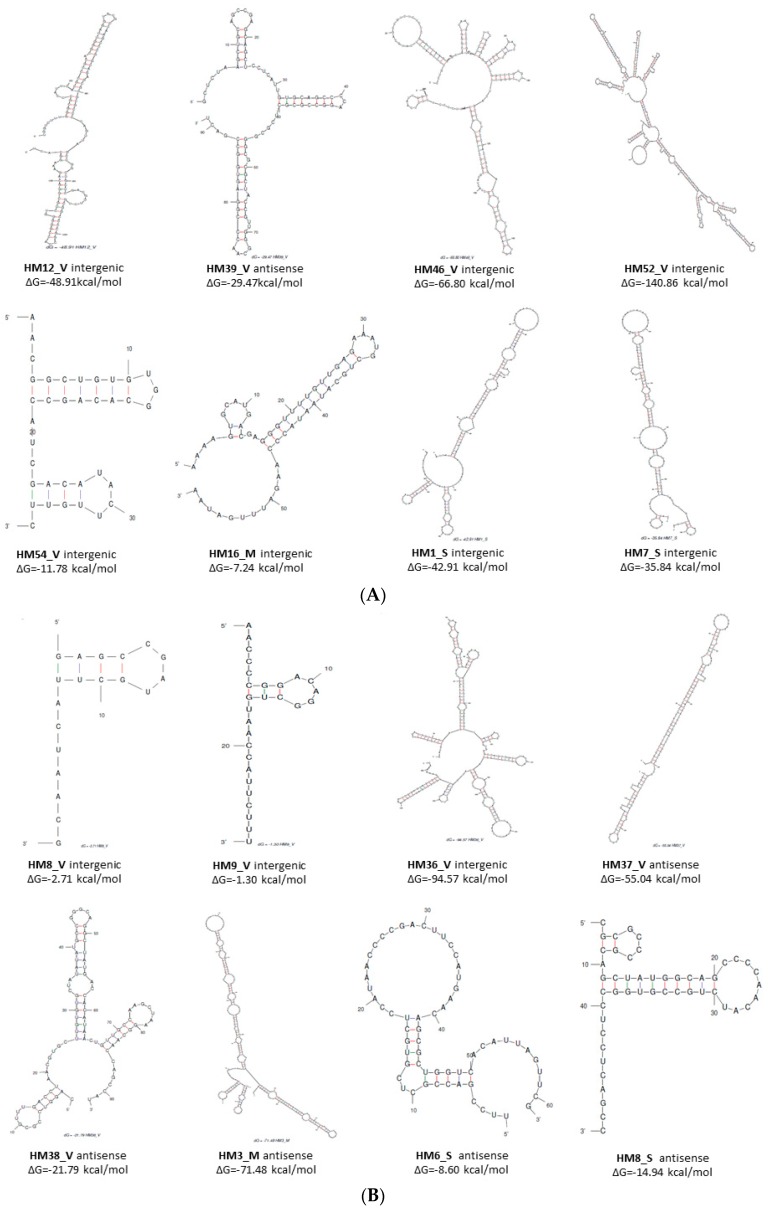
Prediction of the secondary structure of 16 sRNAs with differential expression (nitrate/ammonium) in *Haloferax mediterranei* using Mfold [36]. (**A**) sRNAs overexpressed in nitrate. (**B**) sRNAs overexpressed in ammonium.

Usually, sRNAs are characterised by stable secondary structures. In order to analyse the secondary structure of the 88 putative sRNAs, Mfold software [36] was used with default parameters set and with the temperature modified to 42 °C. The core algorithm predicts a minimum free energy, ΔG°, as well as minimum free energies for folding that must contain any particular base pair. All of the sRNAs were shown to have significant predicted secondary structures since they presented with ΔG < 0. However, only the secondary structures of 16 sRNAs with differential expression according to the nitrogen source (nitrate/ammonium) are shown in Figure 1. Furthermore, most secondary structures of sRNAs revealed highly structured molecules, including more than one hairpin loop and high stability (ΔG < 0). It is noteworthy that the structures of HM39_V, HM54_V, HM38_V, and HM8_S sRNAs present predominant structures with two or three hairpin loops and are similar to the structures of other characterised sRNAs [39,40].

The identification of potential sRNA targets was carried out via a bioinformatic approach using TargetRNA2 [37]. Gene targets were found for 71.59% of the sRNA verified, of which the first five are most likely due to their energy values (Table S8). Taking the five gene targets for each sRNA analysed into account, the most commonly predicted targets correspond to hypothetical proteins with unknown functions. Many examples of transcriptional regulators such as the ArsR family, nitrogen regulatory PII protein, transporters (including metal and ATP-binding cassette (ABC) transporters), proteins related to RNA (i.e., H/ACA RNA-protein complex component Gar1, ribonuclease P subunit p30, 50S ribosomal protein L24e, 50S ribosomal protein L37Ae, DNA-directed RNA polymerase subunit B″, DNA-directed RNA polymerase subunit F, 30S ribosomal protein S8P, etc.), and proteins related to DNA metabolism (i.e., DNA lyase, DNA ligase, DNA double-strand break repair protein mre11, DNA primase large subunit) were also identified. To go in depth with this analysis, the *cis*-encoded sRNAs antisense of characterised ORF were analysed using IntaRNA [38] (Table 3). The results show that nine of the 17 *cis*-encoded sRNAs analysed present high interaction energy and full complementarity with the ORF where they were identified. The combination of these software programs has allowed the identification of possible gene targets for more than 70% of the 88 possible sRNAs identified by RNA-Seq. However, there are still 26 sRNAs whose possible targets could not be found with the software employed.

#### 3.3.3. Conservation of small RNAs Verified in *H. mediterranei*

**Figure 2:**
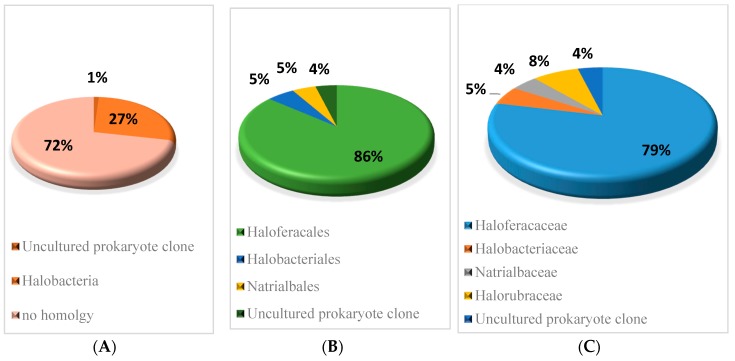
Conservation percentage of the sequences of sRNAs across different (**A**) classes, (**B**) orders, and (**C**) families. BLASTn, homology (*E* value 10 × 10^−6^); query covered at least 80% and sequence identity covered at least 60%.

The sequence conservation of the sRNAs predicted in *H. mediterranei* was analysed using BLASTn [25], comparing each sequence to all sequenced archaeal genomes (*E* value 10 × 10^−6^). Only hits with a nucleotide identity higher than 60% combined with a coverage between the query and subject sequence higher than 80% were considered as conserved. The results of homology are shown in Figure 2 and Table S7. It was found that 27% of verified sRNAs present conserved sequences in other Halobacteria, mainly in the Haloferacales order (86%) and Haloferacaceae family (79%). The remaining 63 sRNAs showed no sequence homology with any other Archaea. 

### 3.4. Expression Analysis According to the Nitrogen Source

The expression analysis of the sRNAs in *H. mediterranei* according to the nitrogen source was performed using the HT-Seq program, which counts the readings of the sRNAs verified by RNA-Seq and DESeq2, which carries out the differential expression analysis (nitrate/ammonium). From the 88 candidate sRNAs analysed, 16 sRNAs met the statistical criteria with a *p*-value and *p*-adj lower than 0.02 and 0.05, respectively (Table 4). These sRNAs show differences in their expression pattern. Eight of them are overexpressed in the presence of nitrate as a sole nitrogen source (log_2_-fold-change between 0.519 and 8.699), whereas the other eight present a decrease in their transcriptional level in presence of ammonium (log_2_-fold-change between −0.478 and 1.325).

### 3.5. Validation of Expression of Small RNAs Using Reverse Transcription Polymerase Chain Reaction

**Figure 3:**
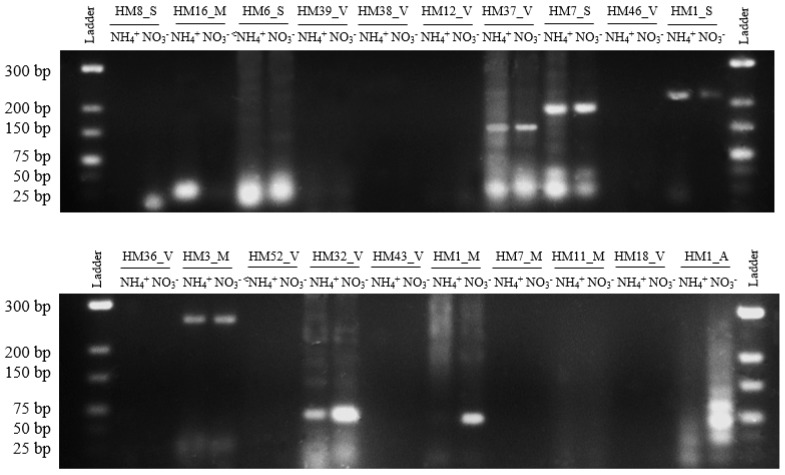
Reverse transcription polymerase chain reaction (RT-PCR) validation of 20 sRNAs. Electrophoresis of PCR products of 20 sRNAs on 3% agarose gel. Lanes 1 and 12: GeneRuler Low Range DNA Ladder (Thermo Scientific, Waltham, MA, USA). Lanes 2–11: PCR products of sRNAs.

To confirm the presence of 20 sRNAs predicted by RNA-Seq we used RT-PCR [41,42,43,44]. Of 20 predicted sRNAs tested, nine (40%) gave positive results of the expected size as shown in Figure 3. These PCR products were subsequently verified using Sanger sequencing. This proportion of successfully validated sRNAs (45%) agrees with the results obtained in other works, where validation is often successful around 40–50% [39,42].

The majority of sRNAs (HM6_S, HM37_V, HM7_S, HM1_S, HM3_M, and HM32_V) are expressed in both conditions, in the presence of nitrate and ammonium. However, three sRNAs show a different expression pattern according to the nitrogen source; HM8_S and HM1_M sRNAs are expressed exclusively in the presence of nitrate and HM16_M sRNA is expressed in the presence of ammonium.

## 4. Discussion

Two different approaches, bioinformatic and RNomic, have allowed the identification of 88 sRNAs in *H. mediterranei*. Most of them are located on the chromosome, as in the case of *H. volcanii.* However, there is a difference between these two halophilic microorganisms. *H. mediterranei* sRNAs are predominantly intergenic (*trans*-encoded sRNA), whereas *H. volcanii* sRNAs are mostly antisense instead [28,45]. 

The 88 sRNAs verified by RNA-Seq were analysed using different strategies to obtain further information about their characteristics and their possible involvement in different cellular processes. Some of the predicted secondary structures of these sRNAs remain similar to other sRNA structures which have been analysed in detail (Figure 1) [39,40]. According to Gaimster et al. [39], these results are significant because they imply that many of these sRNAs could have the potential to form complex conformations like those commonly associated with many other directly-acting RNA transcripts, including known bacterial sRNAs. Moreover, additional molecular mechanisms of sRNA functions in bacteria have been described, including the destabilisation or stabilisation of the target mRNAs. These actions are conducted via specific binding to a protein or full complementarity to their target mRNA [5]. Thus, putative gene targets of all the sRNAs candidates in *H. mediterranei* were predicted using TargetRNA2 [37]. As it has been observed in other works [39], the results obtained reveal that the most commonly predicted targets matched to hypothetical proteins. This information could potentially be useful in eventually assigning a function to these unknown proteins. It has also been identified to target some transcriptional regulators such as the ArsR family, as well as the nitrogen regulatory PII protein. Interestingly, the expression of the ArsR and PII proteins in *H. mediterranei* is closely related to the nitrogen source [24], so the existence of a regulatory network where the sRNAs act by activating or repressing the expression of these proteins is highly likely. In other bacterial studies, it has been observed that global regulators are subject to regulation by multiple Hfq-dependent sRNAs [46]. In the case of domain Archaea, similar interactions have been described between sRNAs and Like Sm (Lsm) homologous proteins, instead of the bacterial Hfq [47]. *H. mediterranei* contains one gene encoding the Lsm1 protein (HFX_2733) in its genome that shares 99% sequence identity with the *H. volcanii* RNA-binding Lsm protein (*E* value 6 × 10^−51^). These results suggest that the interaction between Lsm proteins and sRNAs and their participation in the RNA metabolism are also possible in *H. mediterranei.* Transporters are also included in the different targets predicted, specifically metal and ABC transporters, which have been observed in other analyses of bacterial sRNAs such as *Paracoccus denitrificans* [39] and *Ruegeria pomeroyi* [48], as well as in Archaeal sRNA such as *S. solfataricus* [49] and *H. volcanii* [50]. Based on these results, it could be possible that the regulation of transporters is not a characteristic only of the Bacteria domain. This suggests that antisense sRNA might be a common regulatory mechanism for such genes. It has also been identified as a target of different proteins related to RNA and DNA metabolism, which support the concept that sRNAs play an essential role in transcriptional and post-transcriptional regulation. Furthermore, analysis of co-purification with ribosomal protein L7Ae in *S. solfataricus* allowed for the identification of one sRNA in that microorganism which suggests a direct interaction of these proteins with sRNAs [51]. In parallel, the *cis*-encoded sRNAs antisense of ORF characterised in *H. mediterranei* were also analysed using IntaRNA [38]. This analysis showed positive results in nine of the 17 *cis*-encoded sRNAs. Four of them were present in only one target and the other five could interact with other possible targets. Most sRNAs that did not exhibit interaction with RNA using IntaRNA did exhibit interaction with other mRNAs using TargetRNA2. Therefore, it is likely that some sRNAs are specific to a gene, while others have a broader range of action, being involved in several metabolic pathways (Table S8). Only 26 of the 88 sRNAs analysed did not show positive results using either IntaRNA or TargetRNA2. Hence, more than 70% of the 88 sRNAs verified by RNA-Seq present possible target genes using such software. Nevertheless, it is clear that the only way to confirm the interaction between RNA and its target is through experimental validation. Therefore, in future works we will try to characterise the targets of the sRNAs that present a differential expression in the function of the nitrogen source through the interaction with the protein Lsm1, the generation of deletion sRNA mutants, and other analyses (such as overexpression mutants) to demonstrate the influence of these sRNAs on the respective target.

BLASTn [25] analysis reveals that 27% of sRNAs identified in *H. mediterranei* present conserved sequences in other Halobacteria. Consequently, it is likely that these conserved sRNAs may play a conserved role in closely related species. However, despite the fact that the sequences of the sRNAs are slightly conserved in Archaea, this does not imply that they are all true sRNA candidates. The remaining 72% of sRNAs identified showed no sequence homology to any other archaea. According to Babski et al. [5], many sRNA genes of Archaea are not even shared by species of the same genus. Therefore, this 72% of the sRNA could be specific to the halophilic archaea *H. mediterranei.*

The transcript pattern analysis of *H. mediterranei* sRNA according to the nitrogen source demonstrates that 16 sRNA showed higher or lower transcript levels with statistically significant parameters (*p*-value < 0.02 and *p*-adj < 0.05). Of these, eight show overexpression when *H. mediterranei* grows with nitrate as the sole nitrogen source, whereas the other eight show a decrease in their transcriptional level in the presence of ammonium. The majority of sRNAs validated by RT-PCR are expressed in the presence of nitrogen sources nitrate and ammonium. However, HM8_S and HM16_M sRNAs are expressed exclusively in the presence of nitrate and ammonium, respectively. Curiously, although the HM1_M sRNA transcript level does not show differences in the RNA-Seq analysis according to the nitrogen source, RT-PCR results clearly reveal that this sRNA is only expressed in the presence of nitrate (Figure 3). 

Interestingly, some of the sRNAs which show differences in their expression pattern according to the nitrogen source possibly have target genes whose expression also depends on the nitrogen source. HM8_S, which is overexpressed in nitrate as the nitrogen source, has the protein glutamate dehydrogenase as a possible target (Table 3). Glutamate dehydrogenase is underexpressed in nitrate’s presence. Therefore, this sRNA could be involved in the repression of this key enzyme in the metabolism of nitrogen. Moreover, two sRNAs (i.e., HM7_S and HM54_V) are slightly more expressed in the presence of nitrate. Curiously, their possible targets are transcriptional regulators belonging to the ArsR family. Previous results revealed that the expression of different ArsR proteins depends on the nitrogen source [24]. Therefore, sRNAs could also be involved in the regulation of the expression of these transcriptional regulators, and it could be an example of a gene regulatory network related to nitrogen assimilation. Finally, HM1_A sRNA is expressed in the presence of ammonium as a nitrogen source, and it has been found to be a possible target of the *amt1* gene (Table 3), which encodes an ammonium transporter. Different studies performed with *H. mediterranei* have confirmed that ammonium transporters are expressed in the presence of nitrate or under nitrogen starvation [24]. Hence, this sRNA could also be related to the regulation of Amt transporters expression and, consequently, be adjusting the uptake of ammonium from the medium. More work is needed to confirm these hypotheses and to find out the role of these sRNAs in nitrogen metabolism.

## 5. Conclusions

This work, focused on identifying sRNAs involved in nitrogen assimilation, has also increased the knowledge about sRNAs in the domain Archaea. Specifically, 88 sRNAs have been identified in *H. mediterranei* using bioinformatic and RNomic approaches, some of which show different expression patterns depending on the nitrogen source and/or present genes involved in the nitrogen assimilation as a potential gene target. This data suggests that some of these sRNAs could be related to the regulation of nitrogen assimilation, being able to constitute an important gene regulatory network which involves enzymes, transporters, and transcriptional regulators in this metabolism. Undoubtedly, this work constitutes an excellent starting point to elucidate the role of these sRNAs in the nitrogen metabolism of haloarchaea. 

## Figures and Tables

**Table 1:** Preliminary library of 295 sRNAs in *H. mediterranei* obtained by a bioinformatic approach based on candidate small RNAs of other species.

Reference Species	Number of Candidate sRNAs	Identity (%)	Number of Mismatches	Number of Gaps	*E*-Value
*H. volcanii*	116	74.19–100	<30	<11	<0.05
*M. mazei*	55	82.86–100	<5	<2	<1
*S. solfataricus*	47	95.83–100	<3	<3	<1
*A. fulgidus*	74	73.28–100	<30	<4	<1

**Table 2:** Compilation of positive predicted sRNAs in *H. mediterranei* using RNA-Sequencing.

sRNA Name	Size sRNA (pb)	Position (Start-Stop)	Localisation *H. Med*	Strand	Classification	Gene Environment
HM1_V	131	2947735-2947604	CHR	-	Intergenic	HFX_3032 (gbp3)/HFX_0001(cdc6A-1)
HM3_V	64	602004-601940	CHR	-	Intergenic	HFX_0639/HFX_640 (aspS)
HM4_V	143	2374110-2374253	CHR	-	Intergenic	HFX_2271(htr18-1)/HFX_2270
HM5_V	46	576412-576366	CHR	-	Intergenic	HFX_0609(cdc6C)/HFX_0610
HM6_V	117	401997-401880	CHR	-	Intergenic	HFX_0430(ilvA-1)/HFX_0431
HM8_V	18	310608-310590	CHR	-	Intergenic	HFX_0331(tRNA)/HFX_0332 (rpoH)
HM10_V	42	1913258-1913216	CHR	-	Intergenic	HFX_2748(rpl7AE)/HFX_2747
HM12_V	130	1928092-1927962	CHR	-	Intergenic	HFX_2734/HFX_2733 (lsm1)
HM15_V	113	2841808-2841695	CHR	-	Intergenic	HFX_1820(rRNA)/HFX_2934
HM17_V	81	136329-136248	CHR	-	Intergenic	HFX_0140 (tRNA)/HFX_0141
HM19_V	111	2175910-2175799	CHR	-	Intergenic	HFX_2478/HFX_2477(tRNA)
HM20_V	104	1737798-1737694	CHR	-	Intergenic	HFX_1813/HFX_1814
HM24_V	96	440309-440213	CHR	-	Intergenic	HFX_0475/HFX_0476
HM25_V	90	2584538-2584448	CHR	-	Intergenic	HFX_2084(ydjP-1)/HFX_2083
HM26_V	102	1737680-1737578	CHR	-	Intergenic	HFX_1813/HFX_1814
HM28_V	77	1298728-1298651	CHR	-	Intergenic	HFX_1375(aroA)/HFX_1377
HM30_V	160	248344-248184	CHR	-	Intergenic	HFX_0263/HFX_0264
HM35_V	168	2385624-2385456	CHR	-	Antisense	HFX_2256
HM36_V	262	27244-26982	CHR	-	Intergenic	HFX_0025/HFX_0026
HM38_V	91	2836827-2836736	CHR	-	Intergenic	HFX_1823/HFX_1822
HM39_V	90	2685508-2685418	CHR	-	Antisense	HFX_1980 (abc22A)
HM42_V	58	1769424-1769366	CHR	-	Intergenic	HFX_2905/HFX_2904(folP)
HM46_V	200	2756370-2756170	CHR	-	Intergenic	HFX_1903/HFX_1902
HM47_V	124	1816376-1816252	CHR	-	Intergenic	HFX_2862/HFX_2861
HM49_V	38	359173-359135	pHM500	-	Intergenic	HFX_6336/HFX_6337
HM50_V	78	1116972-1116894	CHR	-	Intergenic	HFX_1173 (tRNA)/HFX_1174(tatAE)
HM52_V	457	2020941-2020484	CHR	-	Intergenic	HFX_2638(dkgB)/HFX_2637(pepC)
HM53_V	156	397174-397018	CHR	-	Intergenic	HFX_0425/HFX_0426
HM54_V	72	2591733-2591661	CHR	-	Intergenic	HFX_2076/2075 (tnp4)
HM55_V	33	486185-486152	CHR	-	Intergenic	HFX_0525(mutS)/HFX_0526(livK)
HM56_V	170	2852306-2852136	CHR	-	Intergenic	HFX_2941(pheS)/HFX_2942
HM2_V	52	681041-681093	CHR	+	Intergenic	HFX_0721/HFX_0722
HM7_V	24	310803-310827	CHR	+	Intergenic	HFX_0331 (tRNA)/HFX_0332 (rpoH)
HM9_V	27	1913243-1913270	CHR	+	Intergenic	HFX_2748(rpl7AE)/HFX_2747
HM11_V	29	1927979-1928008	CHR	+	Intergenic	HFX_2734/HFX_2733 (lsm1)
HM13_V	72	1930742-1930814	CHR	+	Intergenic	HFX_2731(purF)/HFX_2730
HM14_V	49	1931964-1932013	CHR	+	Intergenic	HFX_2729/HFX_2728
HM16_V	246	136148-136394	CHR	+	Intergenic	HFX_0140 (tRNA)/HFX_0141
HM18_V	114	2175756-2175870	CHR	+	Intergenic	HFX_2478/HFX_2477(tRNA)
HM21_V	26	27000891-27000917	CHR	+	Intergenic	HFX_1962/HFX_1961
HM22_V	21	2249860-2249881	CHR	+	Intergenic	HFX_2397 (tRNA)/HFX_2396
HM23_V	111	257489-257600	CHR	+	Intergenic	HFX_0274/HFX_0275 (tRNA)
HM27_V	147	581307-581454	CHR	+	Intergenic	HFX_0614(tRNA)/HFX_0615
HM29_V	258	248240-248498	CHR	+	Intergenic	HFX_0263/HFX_0264
HM31_V	125	1824730-1824855	CHR	+	Intergenic	HFX_2852/HFX_2851
HM32_V	102	2342182-2342284	CHR	+	Intergenic	HFX_2304/HFX_2303
HM33_V	161	221020-221181	CHR	+	Antisense	HFX_0231 (tfb1-1)
HM34_V	95	1259873-1259968	CHR	+	crRNA	HFX_1335/HFX_1336
HM37_V	149	95312-95461	CHR	+	Antisense	HFX_0090 (HemL)
HM40_V	35	1734001-1734036	CHR	+	Antisense	HFX_1808 (ygcJ)
HM41_V	53	2246499-2246552	CHR	+	Antisense	HFX_2401 (xnuC-1)
HM43_V	100	1104687-1104787	CHR	+	Intergenic	HFX_1163 (tRNA)/HFX_1164
HM44_V	38	979793-979831	CHR	+	Intergenic	HFX_1024/HFX_1026
HM45_V	90	809546-809636	CHR	+	Intergenic	HFX_0853/HFX_0854
HM48_V	195	359045-359240	pHM500	+	Intergenic	HFX_6336/HFX_6337
HM51_V	457	2020484-2020941	CHR	+	Intergenic	HFX_2638(dkgB)/HFX_2637(pepC)
HM54_V	35	1299991-1300026	CHR	+	Intergenic	HFX_1375(aroA)/HFX_1377
HM55_V	44	1300050-1300094	CHR	+	Intergenic	HFX_1375(aroA)/HFX_1377
HM1_M	92	2342183-2342275	CHR	+	Intergenic	HFX_2304/HFX_2303 (lactoyglutathione lyase)
HM2_M	34	56050-56016	pHM300	-	Intergenic	HFX_5046 (cyp1_citocromo P450)/ HFX_5047
HM3_M	292	2357250-2357542	CHR	+	Antisense	HFX_2287 (selR)
HM4_M	35	201705-201740	pHM500	+	Antisense	HFX_6177 (nahC2_Antiporter Na/H)
HM5_M	18	14274-14256	pHM500	-	Intergenic	HFX_6013 (nrd-1)/HFX_6014 (sodA)
HM6_M	51	338357-338306	pHM500	-	Antisense	HFX_6316 (csh2-1_CRISPR-associated csh2 family protein)
HM7_M	83	2448865-2448948	CHR	+	Intergenic	HFX_2200/2199 (imd3)
HM8_M	176	804495-804671	CHR	+	Intergenic	HFX_0847/HFX_0848 (epf2-1)
HM9_M	53	1087812-1087865	CHR	+	Antisense	HFX_1142
HM10_M	97	1087972-1087875	CHR	-	Intergenic	HFX_1142/1143
HM11_M	257	709963-709706	CHR	-	Intergenic	HFX_0754 (prot.membr)/HFX_0755
HM12_M	78	862151-862073	CHR	-	Intergenic	HFX_0905/HFX_0906
HM13_M	41	1287240-1287281	CHR	+	Intergenic	HFX_1366/HFX_1367 (htlD-1)
HM14_M	41	1287281-1287240	CHR	-	Intergenic	HFX_1366/HFX_1367 (htlD-1)
HM15_M	153	2508794-2508641	CHR	-	Antisense	HFX_2148 (polysaccharide biosynthesis transporter)
HM16_M	57	2661554-2661497	CHR	-	Intergenic	HFX_2006 (paaJ-1)/HFX_2005 (nasD)
HM1_S	208	2948115-2948323	CHR	+	Intergenic	HFX_3032 (gbp3-1, GTP-binding protein)/HFX_0001 (cell division control protein)
HM2_S	80	412151-412231	CHR	+	Intergenic	HFX_0441 (pyrrolo-quinoline quinone)/HFX_0442 (serine/treonine protein kinase)
HM3_S	98	1691336-1691238	CHR	-	Antisense	HFX_1779 (integrase-like protein)
HM4_S	81	310404-310323	CHR	-	Intergenic	HFX_0329/HFX_0330 (tRNA)
HM5_S	31	1104534-1104503	CHR	-	Antisense	HFX_1161 (tRNA)
HM6_S	60	547749-547689	CHR	-	Antisense	HFX_0587 (ark-6-1, putative signal-transducing histadine kinase)
HM7_S	185	2620188-2620373	CHR	+	Intergenic	HFX_2046/HFX_2045
HM8_S	48	1432982-1432982	CHR	-	Antisense	HFX_1518 (gdhA1, glutamate dehydrogenase NAD (P))
HM1_A	141	99868-100009	CHR	+	Antisense	HM_0095 (amt1-1, ammonium transporter)
HM2_A	482	792936-793418	CHR	+	Antisense	HFX_0839
HM3_A	48	1277917-1277965	CHR	+	Intergenic	HFX_1356/HFX_1357
HM4_A	19	1778748-1778729	CHR	-	Intergenic	HFX_2897 (fumC-1, fumarate hydratase)/HFX_2896 (carbohydrate ATP-binding cassette (ABC) transporter substrate-binding protein)
HM5_A	22	2113156-2113178	CHR	+	Intergenic	HFX_2528/HFX_2529 (gpm, phosphoglycerate mutase)
HM6_A	127	2520322-2520449	CHR	+	Intergenic	HFX_2140 (cdc6H, cell division control protein cdc 6-like protein)/HFX_2139(galE, nucleoside-diphosphate sugar epimerase)

**Table 3:** Analysis of possible targets of antisense sRNAs using IntaRNA [38].

sRNA	sRNA Size (pb)	Gene Target	RNA-Seq Ammonium (+/−)	RNA-Seq Nitrate (+/−)	Target Position	sRNA Position	Energy (Kcal/mol)
HM33_V	161	*tfb1-1*	+	+	513–662	1–150	−247.734
HM35_V	168	*HFX_2256*	+	−	414–468	10–88	−19.201
HM37_V	149	*HemL*	+	+	971–1120	1–149	−238.470
HM39_V	90	*abc22A*	+	+	375–412	28–62	−15.561
HM40_V	35	*ygcJ*	+	+	260–295	1–35	−55.516
HM41_V	53	*xnuC-1*	+	+	583–636	1–53	−86.827
HM3_M	292	*selR*	+	+	316–423	186–292	−162.400
HM4_M	35	*nahC2*, antiporter Na^+^/H^+^	+	+	1151–1186	1–35	−55.151
HM6_M	51	*csh2-1*_CRISPR-associated csh2 family protein	+	+	–	–	–
HM9_M	53	*HFX_1142*	+	+	1–24	1–24	−33.421
HM15_M	153	Polysaccharide biosynthesis transporter	+	+	93–110	115–129	−14.191
HM1_A	141	*amt1-1*, ammonium transporter	+	−	1330–1471	1–141	−229.279
HM2_A	482	HFX_0839	+	+	513–662	301–450	−245.770
HM3_S	98	Integrase-like protein	+	+	17–29	21–33	−9.201
HM5_S	31	tRNA	+	+	47–54	25–32	−1.222
HM6_S	60	*ark-6-1*, putative signal-transducing histadine kinase	+	+	1546–1556	26–37	−12.128
HM8_S	48	*gdhA1*, glutamate dehydrogenase NAD (P)	−	+	230–236	2–8	−6.608

**Table 4:** Summary of the characteristics of sRNAs with differential expression (nitrate/ammonium).

sRNA Name	Localisation	Strand	Position	Size (nt)	Classification	Gene Target ^1^	log_2_ FoldChange	*p*-Value	*p*-adj
HM16_M	CHR	-	2661497-2661554	57	Intergenic	*HFX_1537*, *pgk, HFX_1505*, *trkA, HFX_1789*	8.6985	5.05 × 10^−259^	8.09 × 10^−258^
HM7_S	CHR	+	2620188-2620373	185	Intergenic	*HFX_0627*, *acnA, HFX_1005*, *arsR, HFX_3006*	1.7547	1.29 × 10^−24^	1.03 × 10^−23^
HM52_V	CHR	-	2020941-2020484	457	Intergenic	*hflC*, *HFX_0589*, *HFX_0355*	1.3633	1.06 × 10^−21^	5.96 × 10^−20^
HM46_V	CHR	-	2756370-2756170	200	Intergenic	*HFX_0329, psmA*, *HFX_2148*, *HFX_1154*, *graD5*	1.1966	2.59 × 10^−6^	7.25 × 10^−5^
HM39_V	CHR	-	2685508-2685418	90	Antisense	*ilvB, cbs_4*, *HFX_0847*, *gldA, ligA*	0.8498	0.006	0.0413
HM54_V	CHR	+	2591733-2591661	35	Intergenic	*HFX_0906*, *HFX_1268*, *HFX_0562*, *arsR*, *nfi*	0.6708	0.004	0.0338
HM12_V	CHR	-	1928092-1927962	130	Intergenic	*HFX_1575*, *mutT*, *HFX_2074*, *coxB4*	0.6263	0.003	0.0333
HM1_S	CHR	+	2948115-2948323	208	Intergenic	*livG, HFX_1497*, *HFX_2088*, *HFX_2739*, *nce1*	0.5192	0.0003	0.00086
HM37_V	CHR	+	95312-95461	149	Antisense	*HFX_0565*, *nifS, HFX_1510*, *HFX_0591*	−0.4783	0.0011	0.0158
HM36_V	CHR	-	27244-26982	262	Intergenic	*HFX_2059*, *rpoF*, *apa*	−0.4845	0.0036	0.0332
HM38_V	CHR	-	2836827-2836736	91	Intergenic	*menD, hcpE*, *HFX_1197*, *HFX_0402*	−0.6120	0.008	0.0451
HM3_M	CHR	+	2357250-2357542	292	Antisense	*HFX_2088*, *rnhB, petE*, *HFX_2672*, *HFX_2025*	−0.6247	0.0112	0.0894
HM8_V	CHR	-	310608-310590	18	Intergenic	*no targets*	−0.7387	0.0079	0.0451
HM6_S	CHR	-	547689-547749	60	Antisense	*xthA, dap2*, *HFX_0771*, *HFX_0201*, *yfmJ1*	−0.8373	0.0003	0.00086
HM8_S	CHR	-	1432982-1433030	48	Antisense	*atpl*, *HFX_0366*, *gatD*, *atpF*, *gvpJ*	−0.8791	0.0021	0.00413
HM9_V	CHR	+	1913243-1913270	27	Intergenic	*ppiB*, *HFX_1294*, *HFX_2540*, *ispA*, *HFX_2807*	−1.3253	0.0007	0.0128

^1^ Values of free energy and *p*-value are available in Table S8.

## Supplementary Materials

The following are available online at http://www.mdpi.com/2073-4425/9/2/83/s1. Table S1: Nucleotide sequences of PCR primers for amplifying sRNA genes. Table S2: Library of sRNAs obtained after alignment of *H. volcanii* sRNAs [28] against the *H. mediterranei* genome (*E*-value < 0.05). Table S3: Library of sRNAs obtained after alignment of *M. mazei* sRNAs [17] against the *H. mediterranei* genome (*E*-value < 1). Table S4: Library of sRNAs obtained after alignment of *S. solfataricus* sRNAs [16] against the *H. mediterranei* genome (*E*-value < 1). Table S5: Library of sRNAs obtained after alignment of *A. fulgidus* sRNAs [15] against the *H. mediterranei* genome (*E*-value < 1). Table S6: Sequences of 88 candidates sRNAs identified in *H. mediterranei* by RNA-Seq. Table S7: Conservation of the sequences of sRNAs across different orders, classes, and species. BLASTn, homology (*E* value 10 × 10^−6^, query cover at least 80% and sequence identity at least 60%). Table S8: Prediction of putative gene targets of 88 sRNA in *H. mediterranei* using TargetRNA2 [36].
