# Supplementary material for: Small RNAs of Haloferax mediterranei: Identification and Potential Involvement in Nitrogen Metabolism
Source: Genes (Basel). 2018 Feb 10;9(2):83. doi: 10.3390/genes9020083 (PMC5852579; doi:10.3390/genes9020083)
Supplement: Supplementary file 1 [file genes-09-00083-s001.docx]

**Table S1.** Nucleotide sequences of PCR primers for amplifying sRNA genes.

| **sRNA** | **Forward primer** | **Reverse primer** |
| --- | --- | --- |
| HM8_S | 5’-CGCGCCCGCAGCTATGGC-3’ | 5’- GGCTGAGGAGGCCACGGC-3’ |
| HM16_M | 5’-AAAAGTGCATGACGAGGGTT-3’ | 5’-TTATCAAATCTTGGGTATTATGCA-3’ |
| HM6_S | 5’-TTCCGACCGCTCGTGCTC-3’ | 5’-CGAACTAATGTGACCAGCGCTGT-3’ |
| HM39_V | 5’-GCTCTAAGCTGCAGCCGAGCA-3’ | 5’-AGTCGGCCACTCCGGGTTG-3’ |
| HM38_V | 5’-CAGGTCCGCGTTTGACCTAA-3’ | 5’-ATGGCTGGTTGCCTTAGCTTGG-3’ |
| HM12_V | 5’-TGGTCTTTGGGCGCCCAT-3’ | 5’-AGTGCCTTTCTGCCCGCAGA-3’ |
| HM37_V | 5’-TGCAGCCGTCTGCGCACT-3’ | 5’-GTCTCGGCGAGAAACTGCGG-3’ |
| HM7_S | 5’-CAGCATCGCGACGCAATTACC-3’ | 5’-CCGGCCGGTAGTGGCTGAG-3’ |
| HM46_V | 5’-CCTGCCTTGATCTCATGCAGC-3’ | 5’-GCCCAATTGAGGGAGGGG-3’ |
| HM1_S | 5’-ATCACGTTTCGGGTCCAATT-3’ | 5’-TGTATCTGACACGAACGTAGGGT-3’ |
| HM36_V | 5’-GATGCCCGACCGCGACGGTCGG-3’ | 5’-TGTATCTGACACGAACGTAGGGTCCG-3’ |
| HM3_M | 5’-CGACACTAACCAAGTTTGTTACA-3’ | 5’-AAACGGTTCTGTATCAACTCG-3’ |
| HM52_V | 5’-GCTCTAACTTCGTGTGCTCCATGG-3’ | 5’-GCGGTTGGCTTGGGCAGT-3’ |
| HM32_V | 5’-TGACGACGGCACCGTTCC-3’ | 5’-ATTTGGTCTGAAACGGGACCAGAA-3’ |
| HM43_V | 5’-CCTCGCAAACCTTCGGTTTG-3’ | 5’-GAACGTGACGTCGGCGAGT-3’ |
| HM1_M | 5’-GGCCGATGACGACGGCAC-3’ | 5’-GAAACGGGACCAGAATGGTCGG-3’ |
| HM7_M | 5’-GGAGCACAGAGACGTCGTCC-3’ | 5’-TTTGTCTTCTGAACCGATGAAAAG-3’ |
| HM11_M | 5’-ATGTGTTGGAGTATCCTACCTTCA-3’ | 5’-CCTCAAAATCAAGACAACGC-3’ |
| HM18_V | 5’-AATTTAGATTTGAACCTCGCCGAG-3’ | 5’-GAGCAGAGCGAGGAGTCCGT-3’ |
| HM1_A | 5’-CGTCAGTCCGGACATCGTTC-3’ | 5’-GGGAACGATGTCCGGACTG-3’ |

**Table S2.** Library of sRNAs obtained after alignment of *H. volcanii* sRNAs [28] against the *H. mediterranei* genome (*E* value < 0.05).

| **Original sRNA name** | **Position in *H. volcanii*** (start-stop) | **Size** (pb) | **Alignment position in *H. mediterranei*** | **Localization in *H. med*** | **Identity** (%) | **Mismatches** | **Gaps** |
| --- | --- | --- | --- | --- | --- | --- | --- |
| H75.2 | 1446028-1446247 | 220 | 397793-397776 | CHR | 100 | 0 | 0 |
| H8 | 2745562-2745633 | 72 | 1769463-1769392 | CHR | 100 | 0 | 0 |
| H127 | 249522-249574 | 53 | 571688-571675 | CHR | 100 | 0 | 0 |
| H42 | 2153202-2153299 | 98 | 30642-30628 | CHR | 100 | 0 | 0 |
| H120.2 | 406984-407032 | 49 | 138892-138915 | CHR | 100 | 0 | 0 |
| H100 | 685529-685558 | 30 | 681043-681072 | CHR | 100 | 0 | 0 |
| H226.6 | 408171-408265 | 95 | 396911-396937 | CHR | 100 | 0 | 0 |
| H32 | 2423504-2423539 | 36 | 221063-221077 | CHR | 100 | 0 | 0 |
| H95 | 797446-79752 | 84 | 809546-809623 | CHR | 98.72 | 1 | 0 |
| H3.2 | 2163482-2163629 | 148 | 2342255-2342181 | CHR | 98.67 | 1 | 0 |
| H91 | 936018-936389 | 372 | 979813-980170 | CHR | 98.32 | 6 | 0 |
| H226.21 | 2670192-2670358 | 100 | 1844300-1844252 | CHR | 97.96 | 1 | 0 |
| H225.2 | 1598017-1598113 | 97 | 2841798-2841702 | CHR | 97.94 | 2 | 0 |
| T10 | 2705348-2705587 | 240 | 1816379-1816284 | CHR | 97.92 | 2 | 0 |
| H11.2 | 2705347-2705445 | 99 | 1816379-1816284 | CHR | 97.92 | 2 | 0 |
| H226.13 | 518535-518615 | 81 | 1737781-1737702 | CHR | 97.53 | 1 | 1 |
| H0.2 | 2847079-2847125 | 47 | 2947695-2947728 | CHR | 97.14 | 0 | 1 |
| H44 | 2068154-2068268 | 115 | 2385530-2385500 | CHR | 96.77 | 1 | 0 |
| T5 | 935986-936423 | 438 | 979781-980170 | CHR | 96.42 | 12 | 2 |
| H11.1 | 2078631-2078731 | 101 | 2374290-2374190 | CHR | 96.04 | 4 | 0 |
| H33 | 2392037-2392119 | 83 | 130296-130274 | CHR | 95.65 | 1 | 0 |
| H124 | 311835-311885 | 51 | 310588-310633 | CHR | 95.65 | 2 | 0 |
| H230 | 1080140-1080272 | 133 | 2316259-2316239 | CHR | 95.24 | 1 | 0 |
| H25 | 2499841-2500057 | 217 | 1321738-1321718 | CHR | 95.24 | 1 | 0 |
| H229.10 | 1574908-1574994 | 87 | 1298692-1298651 | CHR | 95.24 | 2 | 0 |
| H106 | 605957-606015 | 59 | 1684637-1684618 | CHR | 95.24 | 0 | 1 |
| H87 | 1070588-1070629 | 42 | 1126824-1126863 | CHR | 95 | 2 | 0 |
| H79 | 1310926-1310985 | 60 | 2246564-2246545 | CHR | 95 | 0 | 1 |
| T22 | 195662-195827 | 166 | 1349976-1349994 | CHR | 95 | 0 | 1 |
| H226.15 | 1733182-1733255 | 74 | 2700907-2700849 | CHR | 94.92 | 2 | 1 |
| T6 | 698893-698988 | 96 | 1758560-1758542 | CHR | 94.74 | 1 | 0 |
| T12 | 2637529-2637627 | 99 | 1116941-1116924 | CHR | 94.74 | 0 | 1 |
| H134 | 97756-97814 | 59 | 111760-111815 | CHR | 94.64 | 3 | 0 |
| H136 | 49539-49652 | 114 | 362209-362192 | CHR | 94.44 | 1 | 0 |
| H129 | 237207-237403 | 197 | 1859647-1859630 | CHR | 94.44 | 1 | 0 |
| H229.2 | 1049090-1049141 | 52 | 1361733-1361749 | CHR | 94.12 | 1 | 0 |
| H88 | 1048996-1049029 | 34 | 1104683-1104716 | CHR | 94.12 | 2 | 0 |
| H30 | 2436137-2436172 | 36 | 461546-461561 | CHR | 94.12 | 0 | 1 |
| H226.12 | 1590152-1590268 | 117 | 1737600-1737663 | CHR | 93.75 | 4 | 0 |
| T1 | 1665733-1665965 | 233 | 2756201-2756391 | CHR | 93.72 | 11 | 1 |
| T25 | 446528-446767 | 240 | 2591725-2591771 | CHR | 93.62 | 2 | 1 |
| H110.1 | 570957-570985 | 29 | 576346-576374 | CHR | 93.1 | 2 | 0 |
| H99 | 702447-702477 | 31 | 698034-698062 | CHR | 93.1 | 2 | 0 |
| H123 | 312017-312072 | 56 | 310770-310825 | CHR | 92.86 | 4 | 0 |
| H17 | 2583137-2583260 | 124 | 1913328-1913220 | CHR | 92.73 | 6 | 2 |
| H60 | 1785150-1785202 | 53 | 2635029-2634989 | CHR | 92.68 | 3 | 0 |
| T20 | 263152-263266 | 115 | 1298649-1298743 | CHR | 92.63 | 7 | 0 |
| H107 | 604153-604256 | 104 | 601967-602020 | CHR | 92.59 | 4 | 0 |
| H77.1 | 155874-155923 | 50 | 2685435-2685460 | CHR | 92.59 | 1 | 1 |
| T24 | 12519-12802 | 284 | 976650-976674 | CHR | 92 | 1 | 1 |
| H226.19 | 2420975-2421160 | 186 | 257587-257539 | CHR | 91.84 | 4 | 0 |
| H226.8 | 518628-518730 | 103 | 1737664-1737605 | CHR | 91.67 | 5 | 0 |
| H21 | 2564677-2564724 | 48 | 1932006-1931960 | CHR | 91.67 | 3 | 1 |
| H19 | 2568338-2568489 | 152 | 1928132-1927981 | CHR | 91.5 | 11 | 2 |
| H226.9 | 575799-575938 | 140 | 581365-581457 | CHR | 91.4 | 8 | 0 |
| T3 | 1522810-1523038 | 229 | 1666940-1667054 | CHR | 91.38 | 9 | 1 |
| H128 | 246735-247070 | 336 | 565766-565745 | CHR | 90.91 | 1 | 0 |
| H131 | 163805-163845 | 41 | 1297814-1297835 | CHR | 90.91 | 2 | 0 |
| H68 | 1619016-1619100 | 85 | 95400-95379 | CHR | 90.91 | 2 | 0 |
| H226.7 | 442805-442946 | 142 | 2584479-2584522 | CHR | 90.91 | 4 | 0 |
| T2 | 1633444-1633489 | 46 | 799219-799198 | CHR | 90.9 | 1 | 1 |
| H229.1 | 1049049-1049090 | 42 | 1298644-1298685 | CHR | 90.48 | 4 | 0 |
| T14 | 2343562-2343711 | 150 | 397094-397075 | CHR | 90 | 2 | 0 |
| H54 | 1831783-1831815 | 33 | 2587374-2587346 | CHR | 90 | 2 | 1 |
| T15 | 2334962-2335169 | 208 | 358990-359028 | pHM500 | 89.74 | 4 | 0 |
| T4 | 1384199-1384394 | 196 | 359028-358990 | pHM500 | 89.74 | 4 | 0 |
| T7 | 486130-486249 | 120 | 486132-486170 | CHR | 89.74 | 4 | 0 |
| T8 | 171872-171985 | 114 | 179614-179642 | CHR | 89.66 | 3 | 0 |
| H3.1 | 2109858-2109941 | 84 | 50887-50869 | CHR | 89.47 | 2 | 0 |
| H225.1 | 1597695-1598068 | 374 | 2842094-2841747 | CHR | 89.17 | 31 | 6 |
| H77.2 | 1334396-1334460 | 65 | 1734008-1734034 | CHR | 88.89 | 2 | 1 |
| H226.2 | 311360-311448 | 89 | 310266-310327 | CHR | 88.71 | 6 | 1 |
| T16 | 2189788-2189860 | 73 | 1174900-1174934 | CHR | 88.57 | 4 | 0 |
| H3.3 | 2822459-2822518 | 60 | 930242-930267 | CHR | 88.46 | 3 | 0 |
| H14 | 2614181-2614214 | 34 | 1333904-1333928 | CHR | 88 | 0 | 2 |
| H226.1 | 121394-121551 | 158 | 136251-136329 | CHR | 87.5 | 9 | 1 |
| H7.4 | 1602956-1603075 | 120 | 2836858-2836735 | CHR | 87.3 | 8 | 6 |
| H226.18 | -2420772-2420893 | 122 | 2175829-2175762 | CHR | 86.96 | 8 | 1 |
| H98 | 706653-706708 | 56 | 702310-702362 | CHR | 86.79 | 7 | 0 |
| H41 | 2160306-2160415 | 110 | 1201472-1201493 | CHR | 86.36 | 3 | 0 |
| H51 | 1891968-1892001 | 34 | 27176-27155 | CHR | 86.36 | 3 | 0 |
| H20 | 2565603-2565697 | 95 | 1930808-1930766 | CHR | 86.05 | 6 | 0 |
| H0.1 | 2847275-2847325 | 51 | 2947881-2947929 | CHR | 86 | 5 | 2 |
| H110.2 | 570620-570683 | 64 | 576021-576082 | CHR | 85.94 | 5 | 3 |
| H118 | 413747-413834 | 88 | 401967-402054 | CHR | 85.71 | 7 | 6 |
| H229.8 | 2303039-2303132 | 94 | 248260-248344 | CHR | 85.56 | 8 | 4 |
| T9 | 2783197_2783326 | 130 | 2852158-2852263 | CHR | 85.19 | 12 | 3 |
| H37.2 | 2357247_2357473 | 227 | 626316-626277 | CHR | 85 | 6 | 0 |
| T23 | 20090_20257 | 168 | 2596725-2596757 | CHR | 84.85 | 5 | 0 |
| T21 | 251337_251565 | 229 | 358990-359028 | pHM500 | 84.62 | 6 | 0 |
| T26 | 424264_42445 | 189 | 358990-359028 | pHM500 | 84.62 | 6 | 0 |
| H229.4 | 1706449_1706550 | 102 | 1298640-1298730 | CHR | 84.62 | 13 | 1 |
| H63 | 1778293_1778341 | 49 | 751312-751350 | CHR | 84.62 | 4 | 2 |
| H226.10 | 1048677_1048757 | 81 | 2175829-2175762 | CHR | 84.29 | 9 | 2 |
| T19 | 34110_34296 | 187 | 359028-358991 | pHM500 | 84.21 | 6 | 0 |
| H74 | 1446315_1446386 | 72 | 1798871-1798908 | CHR | 84.21 | 6 | 0 |
| T27 | 414758_414958 | 201 | 359146-359103 | pHM500 | 84.09 | 4 | 1 |
| T11 | 2653094_2653281 | 188 | 359146-359103 | pHM500 | 84.09 | 3 | 2 |
| T17 | 110730_110916 | 187 | 359103-359146 | pHM500 | 84.09 | 3 | 2 |
| H229.7 | 1953905_1954073 | 169 | 248260-248359 | CHR | 83.96 | 11 | 5 |
| H226.5 | 408064_408178 | 115 | 257539-257594 | CHR | 83.93 | 8 | 1 |
| H226.3 | 311465_311573 | 109 | 440225-440279 | CHR | 83.93 | 7 | 2 |
| H117 | 428357_428513 | 157 | 924418-924388 | CHR | 83.87 | 5 | 0 |
| H49 | 1930097_1930164 | 68 | 1992607-1992572 | CHR | 83.33 | 6 | 0 |
| H225.3 | 2772073_2772138 | 66 | 2842061-2842124 | CHR | 82.81 | 11 | 0 |
| H69 | 1596736_1596810 | 75 | 1744423-1744487 | CHR | 81.82 | 10 | 2 |
| H4 | 2815973_2816050 | 78 | 2464553-2464518 | CHR | 81.58 | 2 | 3 |
| H226.17 | 2263863_2263979 | 117 | 2249881-2249769 | CHR | 81.42 | 11 | 7 |
| H75.1 | 1130816_1130998 | 183 | 1184203-1184265 | CHR | 80.95 | 12 | 0 |
| T13 | 2472606_2472762 | 157 | 2020839-2020740 | CHR | 80 | 20 | 0 |
| H35.2 | 2386033_2386662 | 630 | 1259980-1259884 | CHR | 79.8 | 17 | 3 |
| H55 | 1831561_1831636 | 76 | 1958480-1958431 | CHR | 78.85 | 6 | 5 |
| H81 | 1193394_1193508 | 115 | 1297017-1297118 | CHR | 76.47 | 24 | 0 |
| H23 | 2560307_2560405 | 99 | 1824809-1824718 | CHR | 74.19 | 21 | 2 |
| T18 | 1953740_1953951 | 212 | 1299918-1300061 | CHR | 72.55 | 30 | 11 |

**Table S3.** Library of sRNAs obtained after alignment of *M. mazei* sRNAs [17] against the *H. mediterranei* genome (*E* value < 1).

| **Original sRNA name** | **Position in *M. mazei*** (start-stop) | **Size** (pb) | **Alignment position in *H. mediterranei*** | **Localization in *H. med*** | **Identity** (%) | **Mismatches** | **Gaps** |
| --- | --- | --- | --- | --- | --- | --- | --- |
| sRNA111 | 3020206-3020109 | 97 | 268163-268132 | pHM500 | 87.5 | 4 | 0 |
| sRNA051 | 967401-967341 | 60 | 1287268-1287252 | CHR | 100 | 0 | 0 |
| sRNA143 | 3870097-3870168 | 71 | 64897-64875 | CHR | 91.67 | 1 | 1 |
| sRNA002 | 19212-18973 | 239 | 2076657-2076637 | CHR | 95.24 | 1 | 0 |
| sRNA045 | 840527-840820 | 293 | 201711-201731 | pHM500 | 95.24 | 1 | 0 |
| sRNA072 | 1409359-1409382 | 23 | 1614221-1614238 | CHR | 94.44 | 1 | 0 |
| spRNA42 | 3913870-3914027 | 157 | 321743-321721 | pHM300 | 91.3 | 2 | 0 |
| sRNA160 | 2969755-2969933 | 178 | 419149-419127 | CHR | 91.67 | 0 | 2 |
| sRNA048 | 880183-880258 | 75 | 95954-95972 | pHM100 | 95 | 0 | 1 |
| sRNA155 | 4067977-4067902 | 75 | 250415-250440 | pHM300 | 88.46 | 2 | 1 |
| spRNA06 | 749532-749302 | 230 | 709947-709925 | CHR | 91.67 | 1 | 1 |
| sRNA088 | 2061136-2061100 | 36 | 322542-322528 | CHR | 100 | 0 | 0 |
| spRNA03 | 249071-248814 | 257 | 2246305-2246321 | CHR | 100 | 0 | 0 |
| spRNA11 | 1174850-1175143 | 293 | 862127-862146 | CHR | 95 | 1 | 0 |
| asRNA003 | 193140-193180 | 40 | 1090880-1090897 | CHR | 94.44 | 1 | 0 |
| sRNA082 | 1822377-1822476 | 99 | 1989092-1989107 | CHR | 100 | 0 | 0 |
| sRNA119 | 3193405-3193305 | 100 | 66160-66184 | pHM300 | 88.46 | 2 | 1 |
| asRNA004 | 343417-343312 | 105 | 2448951-2448936 | CHR | 100 | 0 | 0 |
| sRNA072 | 1409359-1409382 | 23 | 1998004-1998017 | CHR | 100 | 0 | 0 |
| sRNA141 | 3847600-3847576 | 24 | 2661562-2661549 | CHR | 100 | 0 | 0 |
| asRNA034 | 3064136-3064185 | 49 | 361166-361180 | CHR | 100 | 0 | 0 |
| asRNA034 | 3064136-3064185 | 49 | 2275007-2274986 | CHR | 90.91 | 1 | 1 |
| sRNA154 | 4065452-4068321 | 131 | 338343-338377 | pHM500 | 82.86 | 5 | 1 |
| asRNA032 | 2936104-2936130 | 26 | 1087863-1087876 | CHR | 100 | 0 | 0 |
| sRNA159 | 2825468-2825442 | 26 | 25548-25532 | pHM300 | 94.12 | 1 | 0 |
| sRNA131 | 3522028-3521977 | 51 | 475020-475034 | CHR | 100 | 0 | 0 |
| asRNA048 | 1041288-1041511 | 223 | 165897-334544 | pHM300 | 92 | 1 | 1 |
| sRNA064 | 1175279-1175306 | 27 | 2718045-2718032 | CHR | 100 | 0 | 0 |
| sRNA065 | 1208072-1208045 | 27 | 133229-133249 | pHM500 | 90.48 | 1 | 1 |
| spRNA08 | 1053914-1054068 | 154 | 1051195-1051210 | CHR | 100 | 0 | 0 |
| spRNA08 | 1053914-1054068 | 154 | 1760524-1760539 | CHR | 100 | 0 | 0 |
| sRNA156 | 4069363-4069207 | 156 | 2560155-2560140 | CHR | 100 | 0 | 0 |
| spRNA42 | 3913870-3914027 | 157 | 56062-56035 | pHM300 | 86.21 | 3 | 1 |
| spRNA26 | 2483727-2483786 | 59 | 2342234-2342254 | CHR | 90.48 | 2 | 0 |
| sRNA121 | 3261968-3261908 | 60 | 287775-287801 | pHM300 | 85.71 | 3 | 1 |
| sRNA134 | 3569603-3569769 | 166 | 14269-14291 | pHM500 | 91.3 | 1 | 1 |
| sRNA019 | 282652-282714 | 62 | 361191-361177 | CHR | 100 | 0 | 0 |
| sRNA019 | 282652-282714 | 62 | 2357478-2357461 | CHR | 94.44 | 1 | 0 |
| sRNA019 | 282652-282714 | 62 | 2523064-2523078 | CHR | 100 | 0 | 0 |
| spRNA23 | 2190267-2490330 | 63 | 863678-863697 | CHR | 90.91 | 0 | 2 |
| sRNA093 | 2190331-2190268 | 63 | 863678-863697 | CHR | 90.91 | 0 | 2 |
| sRNA070 | 1312862-1312926 | 64 | 1294504-1294521 | CHR | 94.44 | 1 | 0 |
| sRNA104 | 2867335-2867271 | 64 | 2094186-2094200 | CHR | 100 | 0 | 0 |
| asRNA019 | 1822201-1822231 | 30 | 524325-524341 | CHR | 94.12 | 1 | 0 |
| spRNA21 | 2104951-2105142 | 191 | 2900493-2900511 | CHR | 94.74 | 1 | 0 |
| asRNA011 | 1260553-1260358 | 195 | 287063-287094 | CHR | 84.38 | 4 | 1 |
| asRNA020 | 2128249-2128218 | 31 | 804548-804535 | CHR | 100 | 0 | 0 |
| sRNA035 | 679306-679287 | 19 | 703404-703416 | CHR | 100 | 0 | 0 |
| spRNA05 | 359868-359652 | 216 | 767659-767677 | CHR | 94.74 | 1 | 0 |
| sRNA158 | 1792230-1792303 | 73 | 2508647-2508633 | CHR | 100 | 0 | 0 |
| sRNA110 | 2997835-2997910 | 75 | 2358462-2358442 | CHR | 90.48 | 2 | 0 |
| sRNA098 | 2343494-2343417 | 77 | 29834-29817 | pHM100 | 94.44 | 1 | 0 |
| sRNA126 | 3370460-3370697 | 237 | 246713-246728 | pHM300 | 100 | 0 | 0 |
| spRNA40 | 3759938-3760177 | 239 | 1158971-1158986 | CHR | 100 | 0 | 0 |

**Table S4.** Library of sRNAs obtained after alignment of *S. solfataricus* sRNAs [16] against the *H. mediterranei* genome (*E* value < 1).

| **Original sRNA name** | **Alignment position in *H. mediterranei*** | **Localization in *H. med*** | **Identity** (%) | **Mismatches** | **Gaps** |
| --- | --- | --- | --- | --- | --- |
| AJ786170 | 2948121-2948107 | CHR | 100.00 | 0 | 0 |
| AJ786172 | 2076358-2076376 | CHR | 94.74 | 1 | 0 |
| AJ786172 | 301031-301014 | CHR | 94.74 | 0 | 1 |
| AJ786172 | 628289-628272 | CHR | 94.44 | 1 | 0 |
| AJ786174 | 2843307-2843338 | CHR | 84.85 | 3 | 2 |
| AJ786176 | 412204-412217 | CHR | 100.00 | 0 | 0 |
| AJ786176 | 957644-957628 | CHR | 94.12 | 1 | 0 |
| AJ786176 | 2418227-2418211 | CHR | 94.12 | 1 | 0 |
| AJ786180 | 1214049-1214065 | CHR | 94.12 | 1 | 0 |
| AJ786180 | 1691275-1691262 | CHR | 100.00 | 0 | 0 |
| AJ786183 | 379983-379966 | pHM500 | 94.44 | 1 | 0 |
| AJ786184 | 2166121-2166135 | CHR | 100.00 | 0 | 0 |
| AJ786185 | 455809-455785 | CHR | 88.89 | 1 | 2 |
| AJ786185 | 456040-456016 | CHR | 88.89 | 1 | 2 |
| AJ786185 | 456252-456226 | CHR | 88.89 | 2 | 1 |
| AJ786185 | 1116831-1116846 | CHR | 100.00 | 0 | 0 |
| AJ786185 | 69644-69630 | CHR | 100.00 | 0 | 0 |
| AJ786185 | 136135-136121 | CHR | 100.00 | 0 | 0 |
| AJ786185 | 528897-528867 | CHR | 84.38 | 2 | 3 |
| AJ786185 | 1737843-1737873 | CHR | 84.38 | 2 | 3 |
| AJ786186 | 1221831-1221803 | CHR | 93.10 | 2 | 0 |
| AJ786186 | 310413-310435 | CHR | 95.83 | 0 | 1 |
| AJ786186 | 310515-310537 | CHR | 95.83 | 0 | 1 |
| AJ786186 | 2783180-2783159 | CHR | 95.45 | 1 | 0 |
| AJ786186 | 1116821-1116848 | CHR | 89.29 | 2 | 1 |
| AJ786186 | 396926-396948 | CHR | 91.67 | 1 | 1 |
| AJ786186 | 310334-310316 | CHR | 94.74 | 1 | 0 |
| AJ786186 | 1692464-1692489 | CHR | 88.46 | 2 | 1 |
| AJ786186 | 136145-1361121 | CHR | 88.00 | 2 | 1 |
| AJ786186 | 1103646-1103632 | CHR | 100.00 | 0 | 0 |
| AJ786186 | 1104516-1104502 | CHR | 100.00 | 0 | 0 |
| AJ786186 | 2695354-2695368 | CHR | 100.00 | 0 | 0 |
| AJ786186 | 2783344-2783327 | CHR | 94.44 | 1 | 0 |
| AJ786190 | 2358432-2358416 | CHR | 94.12 | 1 | 0 |
| AJ786191 | 123362-123346 | pHM500 | 100.00 | 0 | 0 |
| AJ786193 | 282405-282390 | CHR | 100.00 | 0 | 0 |
| AJ786193 | 1040944-1040931 | CHR | 100.00 | 0 | 0 |
| AJ786193 | 1320984-1321000 | CHR | 94.12 | 1 | 0 |
| AJ786205 | 2165913-2165926 | CHR | 100.00 | 0 | 0 |
| AJ786207 | 73443-73422 | pHM500 | 90.91 | 2 | 0 |
| AJ786208 | 159311-159288 | pHM300 | 87.50 | 3 | 0 |
| AJ786210 | 547737-547723 | CHR | 100.00 | 0 | 0 |
| AJ786210 | 2620232-2620249 | CHR | 94.44 | 1 | 0 |
| AJ786211 | 159311-159288 | pHM300 | 87.50 | 3 | 0 |
| AJ786214 | 452484-452468 | pHM500 | 94.44 | 0 | 1 |
| AJ786217 | 475717-475731 | pHM500 | 100.00 | 0 | 0 |
| AJ786218 | 1433004-1432986 | CHR | 94.74 | 0 | 1 |

**Table S5.** Library of sRNAs obtained after alignment of *A. fulgidus* sRNAs [15] against the *H. mediterranei* genome (*E* value < 1).

| **Original sRNA name** | **Alignment position in *H. mediterranei*** | **Localization in *H. med*** | **Identity** (%) | **Mismatches** | **Gaps** |
| --- | --- | --- | --- | --- | --- |
| AJ430238 | 627627-627645 | CHR | 94.74 | 1 | 0 |
| AJ430239 | 1101826-1101806 | CHR | 90.48 | 2 | 0 |
| AJ430240 | 1277936-1277918 | CHR | 94.74 | 1 | 0 |
| AJ430246 | 64801-64826 | pHM100 | 92.59 | 1 | 1 |
| AJ430247 | 237-253 | pHM500 | 94.12 | 1 | 0 |
| AJ430251 | 1753784-1753799 | CHR | 100 | 0 | 0 |
| AJ430252 | 1043501-1043488 | CHR | 100 | 0 | 0 |
| AJ430253 | 492710-492697 | pHM500 | 100 | 0 | 0 |
| AJ430256 | 2336010-2335990 | CHR | 90.48 | 1 | 1 |
| AJ430262 | 1868308-1868295 | CHR | 100 | 0 | 0 |
| AJ430267 | 1644097-1644083 | CHR | 100 | 0 | 0 |
| AJ430269 | 1750502-1750628 | CHR | 73.28 | 30 | 4 |
| AJ430269 | 2836996-2836870 | CHR | 73.28 | 30 | 4 |
| AJ430269 | 1519017-1519036 | CHR | 95 | 1 | 0 |
| AJ430269 | 1747756-1747781 | CHR | 88.46 | 3 | 0 |
| AJ430269 | 2839742-2839717 | CHR | 88.46 | 3 | 0 |
| AJ430269 | 2001775-20001793 | CHR | 95 | 0 | 1 |
| AJ430270 | 175334-175311 | pHM300 | 87.5 | 3 | 0 |
| AJ430275 | 2520433-2520419 | CHR | 100 | 0 | 0 |
| AJ430276 | 2233673-2233660 | CHR | 100 | 0 | 0 |
| AJ430277 | 2342966-2342980 | CHR | 100 | 0 | 0 |
| AJ430283 | 487796-487822 | pHM500 | 85.19 | 4 | 0 |
| AJ430284 | 1905416-1905437 | CHR | 95.45 | 1 | 0 |
| AJ430284 | 810052-810077 | CHR | 88.46 | 3 | 0 |
| AJ430284 | 2073972-2073990 | CHR | 94.74 | 1 | 0 |
| AJ430284 | 785742-785756 | CHR | 100 | 0 | 0 |
| AJ430284 | 2046199-2046185 | CHR | 100 | 0 | 0 |
| AJ430284 | 2469737-2469717 | CHR | 90.48 | 2 | 0 |
| AJ430285 | 1778744-1778730 | CHR | 100 | 0 | 0 |
| AJ430288 | 2090417-2090431 | CHR | 100 | 0 | 0 |
| AJ430289 | 1098623-1098605 | CHR | 94.74 | 1 | 0 |
| AJ430289 | 954676-954690 | CHR | 100 | 0 | 0 |
| AJ430290 | 1324578-1324564 | CHR | 100 | 0 | 0 |
| AJ430292 | 2481887-2481908 | CHR | 90.91 | 2 | 0 |
| AJ430293 | 21267-21250 | pHM100 | 94.44 | 1 | 0 |
| AJ430297 | 17657-17674 | CHR | 94.44 | 1 | 0 |
| AJ430297 | 1478228-1478211 | CHR | 94.44 | 1 | 0 |
| AJ430297 | 1496119-1496136 | CHR | 94.44 | 1 | 0 |
| AJ430299 | 723735-723753 | CHR | 94.74 | 1 | 0 |
| AJ430299 | 793262-793248 | CHR | 100 | 0 | 0 |
| AJ430300 | 27868-27881 | pHM500 | 100 | 0 | 0 |
| AJ430301 | 1824450-1824450 | CHR | 90.91 | 1 | 1 |
| AJ430301 | 2784593-2784576 | CHR | 94.44 | 1 | 0 |
| AJ430302 | 2777854-2777841 | CHR | 100 | 0 | 0 |
| AJ430303 | 376885-376866 | CHR | 95 | 1 | 0 |
| AJ430303 | 99855-99872 | CHR | 94.74 | 0 | 1 |
| AJ430303 | 2524979-2524953 | CHR | 85.71 | 3 | 1 |
| AJ430304 | 707894-707881 | CHR | 100 | 0 | 0 |
| AJ430304 | 2735729-2735713 | CHR | 94.12 | 1 | 0 |
| AJ430309 | 899463-899480 | CHR | 94.74 | 0 | 1 |
| AJ430310 | 33808-33821 | CHR | 100 | 0 | 0 |
| AJ430310 | 2530484-2530468 | CHR | 94.12 | 1 | 0 |
| AJ430310 | 2927553-2927566 | CHR | 100 | 0 | 0 |
| AJ430311 | 2560129-2560153 | CHR | 88.46 | 2 | 1 |
| AJ430313 | 1820626-1820594 | CHR | 85.71 | 3 | 2 |
| AJ430313 | 60577-60600 | CHR | 91.67 | 2 | 0 |
| AJ430313 | 508730-508712 | CHR | 94.74 | 1 | 0 |
| AJ430313 | 1605234-1605216 | CHR | 94.74 | 1 | 0 |
| AJ430313 | 211204-211224 | CHR | 90.48 | 2 | 0 |
| AJ430313 | 650317-650284 | CHR | 82.35 | 4 | 2 |
| AJ430313 | 1102908-1102888 | CHR | 90.91 | 1 | 1 |
| AJ430313 | 1530479-1530493 | CHR | 100 | 0 | 0 |
| AJ430313 | 2072820-2072834 | CHR | 100 | 0 | 0 |
| AJ430313 | 2327156-2327182 | CHR | 85.19 | 4 | 0 |
| AJ430313 | 2568379-2568396 | CHR | 94.44 | 1 | 0 |
| AJ430313 | 2856602-2856616 | CHR | 100 | 0 | 0 |
| AJ430315 | 1473972-1473954 | CHR | 94.74 | 1 | 0 |
| AJ430315 | 2576934-2546916 | CHR | 94.74 | 1 | 0 |
| AJ430315 | 2113172-2113158 | CHR | 100 | 0 | 0 |
| AJ430316 | 128367-128382 | pHM300 | 100 | 0 | 0 |
| AJ430317 | 1664117-1664096 | CHR | 90.91 | 2 | 0 |
| AJ430317 | 2865471-2865489 | CHR | 94.74 | 1 | 0 |
| AJ430317 | 154885-154902 | CHR | 94.44 | 1 | 0 |
| AJ430317 | 560375-560358 | CHR | 94.44 | 1 | 0 |

**Table S6.** Sequences of 88 candidates sRNAs identified in *H. mediterranei* by RNA-Seq.

| **sRNA** | **Sequence (5’🡪3’)** |
| --- | --- |
| **HM1_V** | AAGCTCGGCAAGGTGGACTTTGCTTCCCCACCCCCACACGGGAGGGCTGACAA  AGTAGAG GCACCCTCCCAGAGAGTGCTCAATGAGACCTCTGCTGAGTGAGCAC  CTCTGCACGGTGGC TGAAGATTGACC |
| **HM2_V** | ACATGGTCGCACAGACGATGGATCGCGTTCCGCGACGGTACTGAAATTTTTGT |
| **HM3_V** | GCGTGCGCTCAGAGCAGTCTGGCAGTCGCAGACAGCGGCCTCCGCTGCTGCG  GGGTGACC AATGG |
| **HM4_V** | CTGCGAAGCGAAACCTCCCGGCTTCGCAGTCGCCCCTGGTTAGATCCCGCCGT  TAACGGC GCGCCCGGCGAGGGCACGCCGACGGATAGGGAAAGACTTCTTGGT  TGTGGTTGCTCGGCA GCCGAAGGCTCATGACTGAGCTTG |
| **HM5_V** | TACCACGAGTGCTTGACTGCCTGTCTATAGCCCAAAGTTGTGAGTGT |
| **HM6_V** | CGTGCAGTGTGCGCACGTAAGGGGGAAAGGGGGAAAGGATGCAGCTGTCGCC  AACGCGAG ACAGCGGTTGCAGCAGTGCTCTCGGTGGGGCTACCTCTGACACG  CCTCACTGTAGTGC |
| **HM7_V** | GTAGTATCCCGTGACAGCTTCTTCC |
| **HM8_V** | GAGCCGATGCTTACTAACG |
| **HM9_V** | AACCCCGGACAGGCTGTAACCATTCTTT |
| **HM10_V** | GTCGGACAGGCCCCAACTACTTGGAGGAGCGGCATCCCTAAAG |
| **HM11_V** | TCTGGGGGCCACCGGCCCCGGCGTCTTGCA |
| **HM12_V** | TGGTCTTTGGGCGCCCATTCACGCCTGCACGGGTTAGGTGCGCTAAGCCCGGC  AGAGCGT GCGCCCCAGTTAGCTGGCTGCTCGACGTTCTGCGGCCCCGGCCACC  GGGGGTCTGCGGGC AGAAAGGCACT |
| **HM13_V** | ATTTGCGGGAATGCCCGCCCACCTCGTCTCTGCGACCTGCCGGTCGAGACACG  TACTACTACCCGTCTGAGGG |
| **HM14_V** | CAGGTGATTTCCTACATGGACATCACCCACGCACAATCCTTCCGAACACT |
| **HM15_V** | AGGCTTCCTGTACTCCTAAGGCGGGGACGCCATGCGGCGTTCTGCCATAGACTA  CAATCG GGATCATCAAGCCACTGTAGGCTTGCCTACAGTAAGCTTGAGCCTGG  AAACTTG |
| **HM16_V** | TCAGTCGCGTTGCTCCTTCGCGGGCGCTCCTGAGTCCCACTCGGCGGTGCCTCG  TGGGACTCCCGTCGCGCCCTTTTGCGTTTGTGTTGGTCCACCGAGCGACAGCAA  GACGTGTCGAACCGTCCGCGCGGGATTCGAATCAGGGAGCAACTTGTTGCGAC  CGAGGTTCGAATCTCGTCGCGTGAGCAACGCGAACTGAACCTCTATCGCGGCT  GTAGCCTTCTCGCGGGTTACTGGAACTCTTTCT |
| **HM17_V** | GCGCTGCTCTAAGCTTGGAGCCAGCGTTGTTCAACGAGGGACTAAGCTTAGGG  CGCGCCT GCCAAGCTGTGCAGAACGACAG |
| **HM18_V** | AATTTAGATTTGAACCTCGCCGAGACGTGCTCGCTCACGTTGTTCGCTGTGCGC  GACTCGTCTGGTTCAAATCCGCCGTGACCATTTTCGATTCCACGGACTCCTCGC  TCTGCTC |
| **HM19_V** | AAAAGACTAAGGAGCGTTGCTGCTCGCCTCGCTTGCATCACTCGTCTCGCTCCT  CAGGCA CCTTAGCTTTTACCAGTGCCGCCTAAACTTGGTCTGCTCAGCGCGTGT  CGCT |
| **HM20_V** | AACGAACGTTTAGGCCAGCTCGGCAAACTCGTTCAGTCAGCGTCGCGCACTTG  CTTCACT TGCGCAGCAGACTGACTCAAGTTTAGCCCCTCTCGCCTGAAAAGG |
| **HM21_V** | GGTTCAATTCCCGGTCGGCGCACTCCC |
| **HM22_V** | GTTCGAATCTGCCCCAACCCAC |
| **HM23_V** | AGGCTGCGCCGTACGTACTCACGCACGTACTCACGCTCGTTTCACTCGCGTGA  ACCTCAGTCGCTCTGCTCACTTCGTTCGCATCGCTCCCTGGTTCAATTCTCTCG  GTTAA |
| **HM24_V** | ACTCAGCTCTTTATAGACACTCCCGCCCACCTTGGACTGCTGCTCGCTCCCTTC  GGCTCG CTTCAGCCGCACCAAGCTTAGGCGGGCTCGGGTAGTC |
| **HM25_V** | AAAGTGGGCCACTTCCAAGCTTAGTCCCTTGCTTGCGTCACTCACTGGCACCA  AGCTTAG ACTCGGCCGGGTATGACATATATGGGGAAAG |
| **HM26_V** | TCGTCACTTTGCAGGCAGAGCTAGGCCAAACTCGTCGCTCGTTGCGCTTCGCA  ACGCTCA CTCAAGTTTAGCCCCTCTCGCGTGAGGAAAGACTGCGGCGGAC |
| **HM27_V** | CAGTCCGCGAGAGACTTTTGTGTCTCTCGCACACCTGATTCGCTGTGTACGCT  TCACTGAGATTTGAACTAGCGAGCGAACGCAGTGAGCGAGTGGAGTTCAAAT  CCCACCCTCGGCGCTTCTTGCGGCCACAACACTTCGAGCGGCG |
| **HM28_V** | TAGGCCAGACGGTAGCTGCCACCTAACCCATCCTCAACAGCCTAAGTGAGGG  CGGCATTT GCCGCCCTAAGAGAGGAG |
| **HM29_V** | GTGGTTGCGAACCTGAAACACCCAACGCTCGGGATTCATGCGTTGACGAGAC  TCTTGGTCTCGTTCGCCCACCAGAACGCTCCGCGTTCTAGGGACGTCTTCAGG  GCAGGAGGATATCAATTTGATTCCTTGGACTGTGCCTGCTATCGCTTGCATTG  TTTGGGCTGGTGAGGCTGGCTTCGTCAGCGAAACCAGCAGTGTAATTTCGAG  ACGGCCTGTAGACTGTTCAGTAACCGAAGCGTCCGACTAGCACCGGTAG |
| **HM30_V** | GGACTTCTGCAGGGATCTTGCGCCTCGCAAGACCACCCGCTTGCTCTGGTTCT  CAGAGCA GTTGCGTACTTAGGGCTCGCAACCCACAAAGTCCAAGCGTTGGTG  CTGAGGTCAAAGAAG AAACCCTTGGGCAAAGTGGGTCAATGTCTGCTTGGGA  TCAA |
| **HM31_V** | CTCGTGGTCGCGCGGCGACGTGCCGGACTCCCGCGAGTCGCCGCGCGGCGTA  GGTTAGAGCATAGGCGCGCTGGCCGCGGCTCTGTATATGAATCTTCGTTCGGA  AATGGCAGAAAAAGAGGTGTA |
| **HM32_V** | TGGCCGATGACGACGGCACCGTTCCGAACCGAACTAGGCACTCGGTGATGCG  GAGCCCTATGAGTGCCGAGGCCGACCATTCTGGTCCCGTTTCAGACCAAAT |
| **HM33_V** | TCGCGGTCGAGGTCGAGTTTCGTCGCGAATCGCGGCAGGTACTCGGTCGGGT  CAGCGGGCGCGACGGGGAGACCGAGGTCGCGGTTGAGGGCGCGGTAGGCCG  CTTGGTGCTCGGATTCCGTCGATTTCGAGACGTCGCAGACTTCCTCGACGC  TCCGGGAG |
| **HM34_V** | GCCGCGACAACGTTACAGACGAACCCTAGTTGGGTTGAAGCATTGCCTGTC  CCCGTCGTGTAATCAACTCGGAATCGTTACAGACGAACCCTAGTT |
| **HM35_V** | CCTAACCGAGCCGTAGATTGCCCACAAGAAGCCGTAGATAGCCCACAAAG  AAGCCTCGTG GATTGCGCCGCCAAAGAAGCCGTCGTGCCCTTTATGGTGCT  CTGAGTATGCAGAGCCAGC GGCTCTCCTACACTATTACCAAGCAGCTGTCC  TAACAGGCCCCCACCGG |
| **HM36_V** | GATGCCCGACCGCGACGGTCGGGAGCCGTTTTCGACCTGGTTTATGCATAG  CGCGAGGGC AACCAGCGCGAGTGCGGAGCAGAAGCCGCTCACTTGGTCGC  TGGATGGCTCAGGATTGCC TGGTGAGCCAGCGGCCTACGATCGGTTGCCGG  CAGGCCTACCCAGTGTTAGAGGCCATCA TACCTCAAAGTTTCTCAGCTGTCT  GGCCATGATGTTCTTATAACATGGCGCTGAGCCGGA AGCCGGAGTAGCGCC  GAAAAAAC |
| **HM37_V** | TGCAGCCGTCTGCGCACTCGTCGGCGCGGTCGGCCGAGCCGTGGCGGGTGA  AGACGGTCTTGAACATCGAGTCCGTGCCGACGACGGTGTACTCGGGTGCCT  GGTCTTCGAGGATGTCGGTGATGCCGCGCCGCAGTTTCTCGCCGAGAC |
| **HM38_V** | CAGGTCCGCGTTTGACCTAACGTGCTTGTGTGCTATATATGCCGGGCAGGCT  ATGACCAC ATAACTGTTGCCAAGCTAAGGCAACCAGCCAT |
| **HM39_V** | GCTCTAAGCTGCAGCCGAGCAGCTCCTCATTGTGCAGCCCCAGGCCGCGCT  CGCGGGCGC GCTACCGTTGGGCAACCCGGAGTGGCCGACT |
| **HM40_V** | CCACGAACGGCACTCGCTCGGCGTCAGCGACGACTC |
| **HM41_V** | CGTGATTCCGTCCTCGTTGAGCGATTCGAGCAGGTCGTAGAACGCGTCGAC  CGA |
| **HM42_V** | CACCTGCGGCCTTCCCGTGCGCCCATCCCCATGTGCTCACGGTAAGGCCGGG  TATGTCA |
| **HM43_V** | CCTCGCAAACCTTCGGTTTGCTCAGTCCCGGCGAGTCCACTCACTTCCTTTCG  GTCGTTTCACTCCCTCCAGTCGTTCGTGGACTCGCCGACGTCACGTTC |
| **HM44_V** | TGGTTTTCTGCTCCGCGAGTCGAAAGGCCCTTAACAGTC |
| **HM45_V** | TACCTGAGCGCCTCCGGCGTTCCGGCGGCATAGCACGCAGAATGATCGGGA  ATTCTTATCCACGGTCGGTCGCACTCCAAAGAGCAGCCGC |
| **HM46_V** | CCTGCCTTGATCTCATGCAGCCGTCTCTCTCGGGTCAAGGGCACGGGCTAAC  GCCCGTAC TCCTTTCAGGGGGGTGGCAGACTTGTCCACTGGCCCGCGTTCGG  GCCTCAGCCTCTGCCG ACCGCGACCTTGTCTTTGCTCTGGTGAGCTGGGCTG  GCTACTACGCGCGCTTGGCTGGGC ATTCCCCTCCCTCAATTGGGC |
| **HM47_V** | CTGCGAAGCGAAACCTCCCGGCTTCGCAGTCGCCCCTGGTTAAGTCCCGCCG  TGCGGCAC GGCCTCTAAAGGCCGTGCCGTACGAAAGGAAGATGAGCTACTG  ATAGTCAGACTCAGCGA TGCGG |
| **HM48_V** | TGAAACAGTCGCCGGACCAGCAGTTGACGTCCACTTCAGGCCAGCGATCTC  ACGAAGGTGTCGGCGGCGAACTACCGCCGCCGACACGACAGCGTTGCCACT  GCTTGGAGAGTTGTCCCGGTCGGTATTACCGCTCGAACCGGTCGTCAGGTG  GGCGGTTCGCTGGGATCGAGCGGCGAACTGAGAGTGCCTTCCA |
| **HM49_V** | TTATGGCTGGCCCTGTTGAGAGGTTCGTCACCGTTGCGA |
| **HM50_V** | CGAAGAAGGAGCTAAAAAGAGAGGGCCGGTTGCCCCAGCTTGGTGCGCTA  AAAACTAAGC GGGACTTTCTGCAAAGACA |
| **HM51_V** | CGCCAACCGAACCCGTCACCTACAGGCCACCCAGTGGCCTGATTCGAAGTT  TTTGCCTCCCTTTTGACCTCACTTCCGGCCTTGTTCCGAGCCTCGTTTCCGGG  TTCGTTTCCGACGCCTCGAAGGATTGTGTGTCGGCTGCTCCCGAACTGAGTG  AACATATATAGCGAACACTCTCGTACGTCTGAATATGGCAACGGTGGTGGT  GGTGTGCCCACACTGCGGACAGCAAGTCGAATCGGGATACGAAGGAACGG  CCGACTTTGACGGCGTTCGGCATCGGCTCAAACGCGAATATCGCGTGGCAC  GAGAGACCTGCCCGGTTTGCTCGAATGCGTACGACCTGCGGCGGGCCTGAC  TCCGCGCGAGTCGGTTGACTGTCTCGGTGGGTGGCCGAGGGGTCGAACGGT  TCCGTGTTCAGCTATCTCGGATTCGGTACCTCGTGTGCTTCAATCTCG |
| **HM52_V** | GCTCTAACTTCGTGTGCTCCATGGCTTAGGCTCTATCGACTTGTGCCTTGGC  AAGCTGGG GAGCCGGTGGGTGGCTCTGTCAGTTGGCTGAGCGCGCCTCAGT  CCGGGCGGCGTCCAGCA TGCGTAAGCTCGTTTGGCCCGTCCAGAGAGCACG  GTGCGCTATAAGCGCAAACTCGGCTA CGGCTTGCGGCAGTTTCAGCCGGCA  AGGAAGCATAGGGCTAAGCTGAACGACAGGCGTCACACCCGTGTGGTGG  TGGTGGCAACGGTATAAGTCTGCATGCTCTCACAAGCGATATATAC AAGTG  AGTCAAGCCCTCGTCGGCTGTGTGTTAGGAAGCTCCGCAGCCTTTGCTTGGG  CCT TTGCTCCGAGCCTTGTTCCGGCCTTCACTCCAGTTTTCCCTCCGTTTTTG  AAGCTTAGTC CGGTGACCCACCGGACATCCACTGCCCAAGCCAACCGC |
| **HM53_V** | CGCAGGAACGTGTCCCACGCCGGGAGGAGCCTGAGCCATAGCTTCGCAGCC  AACCCCGCC AGCGGCGCCACCGCCAGCAACGGCCGCAGCGGCAGCGCCGG  TACTGTATGCAGTAGCTTG GCGCTTAGCTTGCCTTTCCTTCCCGAATTTAAT  AGGT |
| **HM54_V** | AACGGCTGTGTGGCACAGCCATCGACATACTTGTTC |
| **HM55_V** | CACGAGAGAACAGGTCGTGTCGTAGACCGGCAAATATCCTACCTG |
| **HM56_V** | CACCCAAGTGACGACGACAGAGTCGCCAGCAATAGCTTGCTTGACCTATCT  GACCAGCAT GACTTGGCATAGC |
| **HM57_V** | AGTAGGCCCTGGCCAGTAGCATCATAAAATAGGC |
| **HM58_V** | TACTGAGAGCTTGGACAGTCAGTACCACAGACGCGGTGAGCGTTTGGCCGAT  GGCTGCCC AAGCCTAAAAAGAAAGACCGGCGAACTCCAGCCGCCTGAGGCG  AAGCCGCGCTGGCGCGG GTCGCCTGCTTTGGCTGGGTAAGCCAGTCTGAGCG  GTCAAGCCGCTCCGAA |
| **HM1_M** | GGCCGATGACGACGGCACCGTTCCGAACCGAACTAGGCACTCGGTGATGCGG  AGCCCTATGAGTGCCGAGGCCGACCATTCTGGTCCCGTTTC |
| **HM2_M** | TATCTAAATTAAGTAACTAAACAACTTACTGGCTA |
| **HM3_M** | CGACACTAACCAAGTTTGTTACATGTCGACTAGCGGCAAATTCTCGTCGTTAG  AAGGGTCCTACTGCGCTGAAACGGGGTTTGGTTGAGGACATACCGTCATCGG  ACTCACCGGGCTGTGTTTGTGACTGAAGTCGACGCCACCAACGTCACTCCGA  CTGCACGGTGTCGTTGCTGTCCGTCCGACTACGCGTCGTCGCCGTCTCCGTCC  GCATCTTCGTCCGGTTCGAAGTCGAGCGCGACCGAGTTGATACAGAACCGTT  TGCCCGTCGGTTCCGGGCCGTCGTCGAAGAC |
| **HM4_M** | ACAGGTTTCTGAGAGTCATCCCCGGCACGACGATGC |
| **HM5_M** | CCAACTCACTGACGGACGG |
| **HM6_M** | TTACATCTGAGGCACGGGCAGACGTAGGTCAACCAGTGCCTCCATAGCGAAG |
| **HM7_M** | GGAGCACAGAGACGTCGTCCACACAAAGTTGCTATCCCTGGAACACAGACTG  CAACGCAACTTTTCATCGGTTCAGAAGACAAA |
| **HM8_M** | TAAGCGGGTTTTCCCTGCCTCTTCCCGCAGTCGAGATACCACCGTATTCAGTTC  ACTATGCTCTCTCCGCAGTTGGCCGAGACCGGCACTCCAACATGTGAACCGTC  TCGTGTTAACACTCCTGAGGTAGGGGTTCCCGAGACCTGCCGGCTCAGGACCG  GACGGGTCGCTCGTAAC |
| **HM9_M** | ATACGACGACGGAGACCGCTTCACTGGTCGCCACTCCGGACCGTTCGATTTTAA |
| **HM10_M** | AAGCGGCGTGGAGGTTTAAGACTGACCCAGCAGCGGCGTAACACTGCAAGCC  CCTGTTGT GGACTCAGACGGAGCTTGACTCAGCCAAAGCATAGAA |
| **HM11_M** | CATCATTCAAGTTATCTTTTATAATTTTATTAAAAGTATAATTCCGGTTCAATG  TGTTGG AGTATCCTACCTTCACAACGTACAGAAGACAAGTTAGCTTTACACC  CACTTTATCCTGGA CAGACAACCAAACACAAAATACAGTGAATCGTTTAGAA  AGGTTTTAAGGAGTGACACTGC TTAAGTTTTTAGTGCTCTGTTCTTGGATTAGT  CTAAGTTAACCATACTTTTATTCTGTGC GTTGTCTTGATTTTGAGG |
| **HM12_M** | CGTCCCCCGAGATGAGTACAAAAGTATGCCTAAGCATGACAATCAATATAG  CAAGTTCCA TCTCCAAAAACCGCGACAG |
| **HM13_M** | GGTGGACTGTCTCGGGGGTCATAGTTCCTCCCCCTCTGCCAC |
| **HM14_M** | CACCGTCTCCCCCTCCTTGATACTGGGGGCTCTGTCAGGTGG |
| **HM15_M** | ATAGCCACGCCGTCAACGATGACCTTGCAGAGATCAAGATAACTTATGGG  AAGCTCAAAG AGTCCGCTCGATAGATGAGCCTTAGGCCGGGCCAAAGAGT  TCGTATTAATTTTAGCAGGG CCCCTAGTACCACAGTCCTTAATATAAACAA  AGA |
| **HM16_M** | AAAAGTGCATGACGAGGGTTTTGTTGAGAAATGCTGCATAATACCCAAGAT  TTGATAA |
| **HM1_S** | ATCACGTTTCGGGTCCAATTCGAGCTCAGTTCGACATGGGTCCGGGCCTAA  TTCGAGTCTAGATAAACGTTTCACCCCACTCGAATCTCACTCGATTCGACCA  GAAGTCGATCTCAGCCGCCCGATACGACTGCAAGTTCTCGTTTTTCGTCGAC  GCGCACATCTTGAAATCGAATGCACGTACGGACCCTACGTTCGTGTCAGAT  ACA |
| **HM2_S** | TCACCTCGCGCGACCAAGTTCTCTGCCGTGTCCAGCACGACCCGTCGTCAG  TTTCTCGCAACCCTCGGAGCCGCTGGTCTC |
| **HM3_S** | TAAAGGTAGACTGGCTAAGACTGTGGAGATATCTGGCAGCTTCATCAGGG  CAGCGCGGAG ATAAAGAAGAAGGAACCAAGCGACCAGCATCACGAACAA |
| **HM4_S** | AAGAGATTAAAGTTGAGCAGGTCGCTCTGCAGAGCAGCGGCTCGCCGTGG  CTCACTTTAA ACACTTGCAACCGTCCGCCCTA |
| **HM5_S** | CCTCTTATCACCTGAGCGGCCCTAAACTTGGG |
| **HM6_S** | TTCCGACCGCTCGTGCTCCATAACCCCGACTTCCATGAACAGCGCTGGTCA  CATTAGTTC G |
| **HM7_S** | CAGCATCGCGACGCAATTACCACCACCACCATAGGACACACCACAGAGGG  GTGCGCCGTGGCCACTCGTCGGCAGTCCGCACACCACAACCACACCACACC  GCACCATTGCGGACGCCACGACCGCTTCCTCCCTCTTTCGAGTAAACTGCG  ACGGGTTTTGGTACACTCAGCCACTACCGGCCGG |
| **HM8_S** | CGCGCCCGCAGCTATGGCAGCCCCAACATCTGCCGTGGCCTCCTCAGCC |
| **HM1_A** | CGTCAGTCCGGACATCGTTCCCACCGTCAGTCCGGACATCGTTCCCACCGT  CAGTCCGGACATCGTTCCCACCGTCAGTCCGGACATCGTTCCCACCGTCAG  TCCGGACATCGTTCCCACCGTCAGTCCGGACATCGTTCCC |
| **HM2_A** | GCGTCTGCGCCGTGGTCGTCGCGCCACGCGAACGCGAAGAGCCGACAAGCG  GGGACCCTGTCCAGTTTGCGTCCGTCTTCGGCAACGCCGGTTGCGCCGTCCC  AGTCGGTTCCGTCGGCCCGGAACGTTCGGCTATCGCCGACCGAGTCGAACG  TGAACCCGGGGTTCGAGAAAGCGTGGATTCCGGCGTCGGTGGCGAAGACG  ACTACTGAGTGGCGATCCTCATCCGGAGACTCCTTCCGCATGTCGACCGTGA  CGACGCCACTGGCAGATTCCACCACCGGAAGCGGGACGCCGACTGCGACTC  CCTCGTGTTCAAGCCCGACAACGACCGTCTTGGGGTCGATGTCGTCGAGGTC  GGCGTCCCAGTCGCGGCCGCCAGTTCCTCGGTGTGCCCCGAGACCGTAGCCG  TCCATCTCGAAGTAGTGCTCGTAGGGTTCGAGGTCGTAGTCGATTGGTTCTG  GGTCGTCGCCGTCGCCAGCG |
| **HM3_A** | TCCCCAGAGAAACACCACCCTGCCGTCGTGTTCGTCGTCCAACGGTAAC |
| **HM4_A** | TAGCCCCTAGACGTAACAAG |
| **HM5_A** | ATCTCCCGACGGCCTTTTCTTTC |
| **HM6_A** | GTGTCCCCCTCTCAAACGCACCGACTGCTGTCTTACAACGCGAAAAGCA  TACGAAATCTACTGTCCTTCACCCGAACCAATACCCGACCGTCGTCGTA  AATCCAAACACCCTTCAAAAACCCATATGG |

| **Tabla S7.** Conservation of the sequences of sRNAs across different orders, classes and species. BLASTn [25], homology (*E* value 10e-6 ), query covered at least 80% and sequence identity at least 60%). | | | | | | | |
| --- | --- | --- | --- | --- | --- | --- | --- |
| **sRNA**  **name** | **Class** | **Order** | **Family** | **Species/Strains** | **Query cover** | **Identity** | ***E* value** |
| HM2_V | Halobacteria | Haloferacales | Haloferacaceae | *Hfx. gibbonsii* strain ARA6 | 100% | 96% | 1e-14 |
|  |  |  |  | *Hfx. volcanii* DS2 | 100% | 96% | 1e-14 |
| HM4_V | Halobacteria | Haloferacales | Haloferacaceae | *Hfx. gibbonsii* strain ARA6 | 86% | 92% | 1e-40 |
| HM10_V | Halobacteria | Haloferacales | Haloferacaceae | *Hfx. volcanii* DS2 | 93% | 100% | 5e-11 |
|  |  |  |  | *Hfx. gibbonsii* strain ARA6 | 90% | 100% | 2e-10 |
| HM11_V | Halobacteria | Haloferacales | Haloferacaceae | *Hfx. gibbonsii* strain ARA6 | 100% | 100% | 1e-06 |
| HM12_V | Halobacteria | Haloferacales | Haloferacaceae | *Hfx. gibbonsii* strain ARA6 | 100% | 92% | 5e-44 |
|  |  |  |  | *Hfx. volcanii* DS2 | 100% | 92% | 2e-42 |
| HM15_V | Halobacteria | Haloferacales | Haloferacaceae | *Hfx. gibbonsii* strain ARA6 | 100% | 98% | 5e-48 |
|  |  |  |  | *Hfx. volcanii* DS2 | 100% | 87% | 2e-46 |
| HM17_V | Halobacteria | Haloferacales | Haloferacaceae | *Hfx. volcanii* DS2 | 96% | 88% | 2e-15 |
| HM20_V | Halobacteria | Haloferacales | Haloferacaceae | *Hfx. volcanii* DS2 | 95% | 97% | 4e-39 |
|  |  |  |  | *Hfx. gibbonsii* strain ARA6 | 95% | 96% | 2e-37 |
| HM28_V | Halobacteria | Haloferacales | Haloferacaceae | *Hfx. volcanii* DS2 | 100% | 92% | 1e-21 |
|  |  |  |  | *Hfx. volcanii* DS2 plasmid pHV4 | 100% | 91% | 7e-20 |
| HM32_V | Halobacteria | Haloferacales | Haloferacaceae | *Hfx. volcanii* DS2 | 100% | 89% | 5e-18 |
|  |  |  |  | *Halogeometricum borinquense* DSM 11551 | 100% | 93% | 8e-21 |
| HM33_V | Halobacteria | Haloferacales | Haloferacaceae | *Hfx. gibbonsii* strain ARA6 | 100% | 90% | 2e-49 |
| HM37_V | Halobacteria | Haloferacales | Haloferacaceae | *Hfx. gibbonsii* strain ARA6 | 98% | 90% | 2e-44 |
|  |  |  |  | *Hfx. volcanii* DS2 | 97% | 88% | 6e-39 |
| HM38_V | Halobacteria | Haloferacales | Haloferacaceae | *Hfx. gibbonsii* strain ARA6 | 97% | 89% | 2e-20 |
| HM41_V |  | Halobacteriales | Halobacteriaceae | *Halobacterium* sp. DL1 | 100% | 98% | 8e-17 |
| HM42_V | Halobacteria | Haloferacales | Haloferacaceae | *Hfx. gibbonsii* strain ARA6 | 100% | 100% | 3e-21 |
|  |  |  |  | *Hfx. volcanii* DS2 | 100% | 98% | 1e-19 |
| HM43_V | Halobacteria | Haloferacales | Haloferacaceae | *Hfx. volcanii* DS2 | 92% | 90% | 3e-15 |
|  |  |  |  | *Hfx. gibbonsii* strain ARA6 | 85% | 90% | 2e-12 |
| HM45_V | Halobacteria | Haloferacales | Haloferacaceae | *Hfx. gibbonsii* strain ARA6 | 100% | 97% | 1e-33 |
|  |  |  |  | *Hfx. volcanii* DS2 | 100% | 97% | 1e-33 |
|  |  |  |  | *Halogeometricum borinquense* DSM 11551 | 100% | 91% | 2e-25 |
|  |  |  | Halorubraceae | *Halopenitus persicus* CBA 1233 | 81% | 82% | 5e-07 |
| HM46_V | Halobacteria | Haloferacales | Haloferacaceae | *Hfx. volcanii* DS2 | 81% | 95% | 2e-64 |
|  |  |  |  | *Hfx. gibbonsii* strain ARA6 | 83% | 93% | 1e-61 |
| HM48_V |  |  |  | *Uncultured prokaryote clone DeadSea1_IOBCFE001_09-D06-Fwd genomic sequence* | 99% | 80% | 4e-32 |
| HM53_V | Halobacteria | Haloferacales | Haloferacaceae | *Hfx. gibbonsii* strain ARA6 | 100% | 89% | 5e-45 |
|  |  |  |  | *Hfx. volcanii* DS2 | 100% | 89% | 5e-45 |
| HM54_V | Halobacteria | Haloferacales | Halorubraceae | *Halorubrum lacusprofundi* strain HLS1 plasmid pR1SE2 | 100% | 100% | 5e-09 |
|  |  |  |  | *Halorubrum lacusprofundi* strain ATCC 49239 plasmid pHLAC01 | 100% | 100% | 5e-09 |
|  |  |  |  | *Halorubrum lacusprofundi*  ATCC 49239 chromosome 2 | 100% | 100% | 5e-09 |
|  |  |  |  | *Haloterrigena turkmenica* DSM 5511 plasmid pHTUR01 | 100% | 97% | 2e-07 |
|  |  | Natrialbales | Natrialbaceae | *Natrialba magadii* ATCC 43099 plasmid pNMAG02 | 86% | 100% | 3e-06 |
| HM55_V | Halobacteria | Haloferacales | Haloferacaceae | *Haloquadratum walsbyi* DSM 16790 | 100% | 98% | 5e-12 |
|  |  |  | Halorubraceae | *Halorubrum trapanicum* CBA1232 | 100% | 98% | 5e-12 |
|  |  |  |  | *Halorubrum lacusprofundi* ATCC 49239 | 100% | 96% | 2e-10 |
|  |  | Natrialbales | Natrialbaceae | *Halobiforma lacisalsi* AJ5 | 100% | 93% | 1e-08 |
| HM1_M | Halobacteria | Haloferacales | Haloferacaceae | *Hfx. gibbonsii* strain ARA6 | 80% | 100% | 9e-30 |
|  |  |  |  | *Halogeometricum borinquense* DSM 11551 | 100% | 93% | 2e-20 |
|  |  | Halobacteriales | Halobacteriaceae | *Salinarchaeum sp. Harcht-Bsk1* | 86% | 89% | 1e-17 |
| HM8_M | Halobacteria | Haloferacales | Haloferacaceae | *Hfx. gibbonsii* strain ARA6 | 100% | 95% | 4e-71 |
|  |  |  |  | *Hfx. volcanii* DS2 | 100% | 93% | 2e-64 |
| HM5_S | Halobacteria | Natrialbales | Natrialbaceae | *Haloterrigena daqingensis* strain JX313 | 93% | 100% | 6e-06 |
|  |  |  |  | *Natronobacterium gregoryi* SP2 | 93% | 100% | 6e-06 |
|  |  | Haloferacales | Halorubraceae | *Halomicrobium* mukohataei DSM 12286, | 93% | 100% | 6e-06 |

**Table S8.** Prediction of putative gene targets of 88 sRNA in *H. mediterranei* using TargetRNA2 [37].

| **sRNA** | **Gene** | | **Protein** | **Energy (kcal/mol)** | ***p* value** |
| --- | --- | --- | --- | --- | --- |
| **HM1_V** | *HFX_0349* | | hypothetical protein | -14.24 | 0.001 |
|  | *HFX_2082* | | hypothetical protein | -11.18 | 0.012 |
|  | *pphA* | | serine/threonine protein phosphatase | -9.99 | 0.023 |
|  | *HFX_1710* | | hypothetical protein | -9.56 | 0.029 |
|  | *HFX_2949* | | hypothetical protein | -9.47 | 0.030 |
| **HM2_V** | *gph* | | haloacid dehalogenase superfamily enzyme, subfamily IA | -13.37 | 0.003 |
|  | *metC* | | cystathionine synthase/lyase (cystathionine gamma-synthase, cystathionine gamma-lyase, cystathionine beta-lyase) | -11.84 | 0.008 |
|  | *HFX_1898* | | hypothetical protein | -11.71 | 0.009 |
|  | *HFX_2063* | | hypothetical protein | -11.13 | 0.013 |
|  | *HFX_0097* | | hypothetical protein | -10.1 | 0.022 |
| **HM3_V** | *no targets* | | no targets |  |  |
| **HM4_V** | *no targets* | | no targets |  |  |
| **HM5_V** | *no targets* | | no targets |  |  |
| **HM6_V** | *no targets* | | no targets |  |  |
| **HM7_V** | *HFX_2843* | | hypothetical protein | -11.1 | 0.013 |
|  | *HFX_1707* | | hypothetical protein | -10.12 | 0.022 |
|  | *gph* | | haloacid dehalogenase superfamily enzyme, subfamily IA | -9.58 | 0.029 |
|  | *HFX_2918* | | hypothetical protein | -9.41 | 0.031 |
|  | *phnD1* | | putative phosphonate ABC transporter, periplasmic  phosphonate-binding protein | -9.06 | 0.036 |
| **HM8_V** | *no targets* | | no targets |  |  |
| **HM9_V** | *HFX_1283* | | hypothetical protein | -12.12 | 0.007 |
|  | *trh5* | | transcription regulator | -11.29 | 0.012 |
|  | *HFX_1609* | | hypothetical protein | -10.94 | 0.014 |
|  | *perM* | | putative permease | -10.5 | 0.018 |
|  | *HFX_1165* | | hypothetical protein | -10.06 | 0.023 |
| **HM10_V** | *no targets* | | no targets |  |  |
| **HM11_V** | *no targets* | | no targets |  |  |
| **HM12_V** | *no targets* | | no targets |  |  |
| **HM13_V** | *no targets* | | no targets |  |  |
| **HM14_V** | *HFX_2687* | | hypothetical protein | -9.79 | 0.026 |
|  | *HFX_2985* | | hypothetical protein | -9.59 | 0.028 |
|  | *yqjM2* | | NADH-dependent flavin oxidoreductase | -9.35 | 0.032 |
|  | *HFX_1497* | | hypothetical protein | -9.05 | 0.036 |
|  | *pstS2* | | phosphate ABC transporter periplasmic substrate-binding protein | -8.74 | 0.042 |
| **HM15_V** | *no targets* | | no targets |  |  |
| **HM16_V** | *paaJ* | | acetyl-CoA C-ac(et)yltransferase | -13.53 | 0.003 |
|  | *gcp* | | O-sialoglycoprotein endopeptidase/protein kinase | -13.39 | 0.003 |
|  | *HFX_1816* | | hypothetical protein | -12.17 | 0.007 |
|  | *graD5* | | glucose-1-phosphate thymidylyltransferase | -11.32 | 0.011 |
|  | *mcmB* | | methylmalonyl-CoA mutase, subunit B (cobalamin-binding subunit | -10.94 | 0.014 |
| **HM17_V** | *HFX_2095* | | hypothetical protein | -14.67 | 0.001 |
|  | *ndh* | | NADH dehydrogenase | -9.79 | 0.026 |
|  | *HFX_2127* | | hypothetical protein | -9.67 | 0.027 |
|  | *gckA* | | hydroxypyruvate reductase, glycerate kinase | -9.51 | 0.029 |
|  | *HFX_0171* | | hypothetical protein | -9.37 | 0.032 |
| **HM18_V** | *folA* | | dihydrofolate reductase | -16.94 | 0.000 |
|  | *HFX_2270* | | hypothetical protein | -16.53 | 0.000 |
|  | *HFX_0917* | | hypothetical protein | -16.13 | 0.000 |
|  | *dsbB2* | | disulfide bond formation protein DsbB | -14.94 | 0.001 |
|  | *etfA* | | electron transfer flavoprotein alpha-subunit | -13.77 | 0.002 |
| **HM19_V** | *HFX_2059* | | hypothetical protein | -11.68 | 0.009 |
|  | *rpoF* | | DNA-directed RNA polymerase subunit F | -11.28 | 0.012 |
|  | *apa* | | diadenosine tetraphosphate pyrophosphohydrolase | -9.9 | 0.025 |
| **HM20_V** | *menD* | | 2-oxoglutarate decarboxylase;  2-succinyl-6-hydroxy-2,4-cyclohexadiene-1-carboxylate synthase /  2-succinyl-5-enolpyruvyl-6-hydroxy-3-cyclohexene-1-carboxylate synthase | -9.87 | 0.025 |
|  | *hcpE* | | 2-hydroxyhepta-2,4-diene-1,7-dioate isomerase | -9.81 | 0.026 |
|  | *HFX_1197* | | hypothetical protein | -8.73 | 0.042 |
|  | *HFX_0420* | | hypothetical protein | -8.38 | 0.048 |
| **HM21_V** | *ilvB* | | acetolactate synthase I/II/III large subunit | -14.98 | 0.010 |
|  | *cbs_4* | | CBS domain-containing protein | -13.98 | 0.002 |
|  | *HFX_0847* | | hypothetical protein | -13 | 0.004 |
|  | *gldA* | | glycerol-1-phosphate dehydrogenase (NAD(P)) | -12.38 | 0.006 |
|  | *ligA* | | DNA ligase (NAD) | -12.36 | 0.006 |
| **HM22_V** | *sbcD* | | DNA double-strand break repair protein mre11 | -14.11 | 0.002 |
|  | *abc22P* | | molybdenum ABC transporter permease protein | -12.85 | 0.004 |
|  | *HFX_0809* | | hypothetical protein | -11.73 | 0.009 |
|  | *fhuB* | | ABC-type cobalamin/Iron(III)-siderophore transport systems,  substrate-binding protein | -9.59 | 0.029 |
|  | *HFX_1448* | | hypothetical protein | -9.55 | 0.029 |
| **HM23_V** | *HFX_0329* | | hypothetical protein | -17.77 | 0.000 |
|  | *psmA* | | proteasome subunit alpha | -14.82 | 0.001 |
|  | *HFX_2148* | | polusaccharide biosynthesis transporter | -12.32 | 0.006 |
|  | *HFX_1154* | | hypothetical protein | -11.77 | 0.009 |
|  | *graD5* | | glucose-1-phosphate thymidylyltransferase | -11.63 | 0.009 |
| **HM24_V** | *HFX_1713* | | hypothetical protein | -15.92 | 0.000 |
|  | *HFX_2661* | | hypothetical protein | -12.59 | 0.005 |
|  | *HFX_2915* | | hypothetical protein | -10.99 | 0.014 |
|  | *fabG3* | | 3-oxoacyl-[acyl-carrier protein] reductase | -9.49 | 0.030 |
|  | *HFX_1374* | | type I phosphodiesterase/nucleotide pyrophosphatase | -9.33 | 0.032 |
| **HM25_V** | *no targets* | | no targets |  |  |
| **HM26_V** | *no targets* | | no targets |  |  |
| **HM27_V** | *hflC* | | SPFH domain, Band 7 family protein | -10.3 | 0.020 |
|  | *HFX_0589* | | hypothetical protein | -8.56 | 0.045 |
|  | *HFX_0355* | | hypothetical protein | -8.52 | 0.046 |
| **HM28_V** | *ppA* | | inorganic pyrophosphatase | -8.37 | 0.048 |
| **HM29_V** | *HFX_1998* | | hypothetical protein | -10.81 | 0.015 |
| **HM30_V** | *ilvB* | | acetolactate synthase I/II/III large subunit | -15.71 | 0.000 |
|  | *amt2* | | ammonium transporter | -13.04 | 0.004 |
|  | *pphA* | | serine/threonine protein phosphatase | -12.38 | 0.006 |
|  | *tnp3* | | transposase | -12.03 | 0.007 |
|  | *pyrF* | | orotidine-5'-phosphate decarboxylase | -11.72 | 0.009 |
| **HM31_V** | *HFX_2598* | | hypothetical protein | -13.32 | 0.003 |
|  | *HFX_1102* | | ribonuclease P subunit p30 | -11.27 | 0.012 |
|  | *HFX_1736* | | hypothetical protein | -11.01 | 0.014 |
|  | *yidJ* | | arylsulfatase | -10.54 | 0.018 |
|  | *HFX_1994* | | hypothetical protein | -10.44 | 0.019 |
| **HM32_V** | *ftsZ3* | | cell division protein ftsZ | -17.94 | 0.000 |
|  | *pyrE* | | orotate phosphoribosyltransferase | -16.51 | 0.000 |
|  | *rfcB* | | replication factor C large subunit | -15.37 | 0.000 |
|  | *grxC* | | glutathione S-transferase domain-containing protein | -12.56 | 0.005 |
|  | *rpl24A* | | 50S ribosomal protein L24e | -11.07 | 0.013 |
| **HM33_V** | *no targets* | | no targets |  |  |
| **HM34_V** | *ppiB* | | peptidyl-prolyl cis-trans isomerase B (cyclophilin B) | -18.67 | 0.000 |
|  | *HFX_1294* | | putative phage integrase | -16.37 | 0.000 |
|  | *HFX_2540* | | hypothetical protein | -16.33 | 0.000 |
|  | *ispA* | | multifunctional long-chain (E)-prenyl diphosphate synthase | -13.83 | 0.002 |
|  | *HFX_2807* | | hypothetical protein | -13.45 | 0.003 |
| **HM35_V** | *fapE1* | | flagella-related protein E | -10.92 | 0.014 |
|  | *acrR* | | DNA binding protein putative transcriptional regulator | -10.66 | 0.017 |
|  | *HFX_0034* | | hypothetical protein | -10.65 | 0.017 |
|  | *dps* | | DNA-binding ferritin-like protein (oxidative damage protectant) | -10.03 | 0.023 |
|  | *HFX_2341* | | hypothetical protein | -10.03 | 0.023 |
| **HM36_V** | *hcpB* | | halocyanin hcpB | -13.01 | 0.004 |
|  | *HFX_1569* | | hypothetical protein | -8.71 | 0.042 |
| **HM37_V** | *no targets* | | no targets |  |  |
| **HM38_V** | *no targets* | | no targets |  |  |
| **HM39_V** | *no targets* | | no targets |  |  |
| **HM40_V** | *no targets* | | no targets |  |  |
| **HM41_V** | *no targets* | | no targets |  |  |
| **HM42_V** | *no targets* | | no targets |  |  |
| **HM43_V** | *HFX_0351* | | hypothetical protein | -18.27 | 0.000 |
|  | *gcvP1* | | glycine dehydrogenase subunit 1 | -17.89 | 0.000 |
|  | *HFX_0917* | | hypothetical protein | -16.15 | 0.000 |
|  | *HFX_2335* | | GCN5-related N-acetyltransferase | -15.81 | 0.000 |
|  | *HFX_0559* | | hypothetical protein | -15.31 | 0.001 |
| **HM44_V** | *secG* | | preprotein translocase subunit SecG | -9.11 | 0.036 |
| **HM45_V** | *aat1* | | pyridoxal phosphate-dependent aminotransferase  (histidinol-phosphate aminotransferase-like protein /  aspartate aminotransferase | -17.15 | 0.000 |
|  | *HFX_1316* | | hypothetical protein | -14.67 | 0.001 |
|  | *pstC5* | | phosphate ABC transporter permease | -13.87 | 0.002 |
|  | *rbnBN1* | | ribonuclease BN-like protein | -12.05 | 0.003 |
|  | *usp* | | stress response protein | -11.75 | 0.007 |
| **HM46_V** | *no targets* | | no targets |  |  |
| **HM47_V** | *no targets* | | no targets |  |  |
| **HM48_V** | *sodA* | | Fe-Mn family superoxide dismutase | -9.76 | 0.026 |
|  | *HFX_6114* | | hypothetical protein | -8.73 | 0.042 |
|  | *wecB* | | UDP-N-acetylglucosamine 2-epimerase | -8.33 | 0.049 |
| **HM49_V** | *HFX_6217* | | hypothetical protein | -12.8 | 0.004 |
|  | *HFX_6204* | | CopG family ribbon-helix-helix transcription regulator | -9.58 | 0.029 |
|  | *HFX_6436* | | hypothetical protein | -8.47 | 0.047 |
| **HM50_V** | *HFX_2090* | | hypothetical protein | -13.6 | 0.002 |
|  | *HFX_2526* | | hypothetical protein | -13.38 | 0.003 |
|  | *arsR (HFX_2192)* | | putative transcriptional regulator, ArsR family | -13.26 | 0.003 |
|  | *HFX_2611* | | hypothetical protein | -12.95 | 0.003 |
|  | *HFX_24171* | | hypothetical protein | -12.66 | 0.004 |
| **HM51_V** | *HFX_2528* | | hypothetical protein | -24.12 | 0 |
|  | *HFX_1354* | | hypothetical protein | -20.84 | 0 |
|  | *HFX_1019* | | hydrolase-like protein | -20.2 | 0 |
|  | *HFX_2800* | | sialidase-1 | -20.01 | 0 |
|  | *gst1* | | glutathione S-transferase | -19.71 | 0 |
| **HM52_V** | *HFX_1589* | | hypothetical protein | -16.07 | 0 |
|  | *cbaA* | | cytochrome c oxidase polypeptide I | -11.78 | 0.009 |
|  | *mscS* | | mechanosensitive ion channel | -11.1 | 0.013 |
|  | *sec11* | | signal sequence peptidase | -10.76 | 0.016 |
|  | *lig* | | DNA ligase (ATP) | -10.73 | 0.016 |
| **HM53_V** | *no targets* | | no targets |  |  |
| **HM54_V** | *dppA* | | ABC-type dipeptide/oligopeptide/nickel transport system,  substrate binding protein | -14.8 | 0.001 |
|  | *rpl37AR* | | 50S ribosomal protein L37Ae | -11.81 | 0.008 |
|  | *htr31* | | transducer protein htr15 | -10.59 | 0.017 |
|  | *HFX_0523* | | hypothetical protein | -9.64 | 0.028 |
|  | *HFX_2374* | | ATPase | -9.63 | 0.028 |
| **HM55_V** | *sdhA* | | succinate dehydrogenase, subunit A (flavoprotein) | -17.15 | 0 |
|  | *HFX_2369* | | NAD dependent epimerase/dehydratase family protein | -15.18 | 0.001 |
|  | *HFX_0439* | | hypothetical protein | -14.56 | 0.001 |
|  | *HFX_1569* | | hypothetical protein | -14.08 | 0.002 |
|  | *gvpN* | | gas-vesicle operon protein gvpN | -13.68 | 0.002 |
| **HM56_V** | *no targets* | | no targets |  |  |
| **HM57_V** | *HFX_0906* | | phage PhiH1 repressor protein | -10.82 | 0.015 |
|  | *HFX_1268* | | hypothetical protein | -10.62 | 0.017 |
|  | *HFX_0562* | | hypothetical protein | -10.42 | 0.019 |
|  | *arsR (HFX_0459)* | | putative transcriptional regulator, ArsR family | -10.14 | 0.022 |
|  | *nfi* | | endonuclease V (deoxyinosine 3'endonuclease) | -9.36 | 0.032 |
| **HM58_V** | *no targets* | | no targets |  |  |
| **HM1_M** | *ftsZ3* | | cell division protein ftsZ | -17.94 | 0 |
|  | *pyrE* | | orotate phosphoribosyltransferase | -16.51 | 0 |
|  | *rfcB* | | replication factor C large subunit | -15.37 | 0 |
|  | *pri2* | | DNA primase large subunit | -13.62 | 0.002 |
|  | *HFX_0225* | | hypothetical protein | -13.53 | 0.002 |
| **HM2_M** | *no targets* | | no targets |  |  |
| **HM3_M** | *HFX_2088* | | hypothetical protein | -16.23 | 0 |
|  | *rnhB* | | ribonuclease HII | -15.42 | 0 |
|  | *petE* | | halocyanin precursor-like protein | -12.37 | 0.006 |
|  | *HFX_2672* | | hypothetical protein | -12.26 | 0.006 |
|  | *HFX_2025* | | hypothetical protein | -12.21 | 0.006 |
| **HM4_M** | *pstS* | | phosphate ABC transporter substrate binding protein | -12.54 | 0.005 |
|  | *sodA* | | Fe-Mn family superoxide dismutase | -12.45 | 0.006 |
|  | *yvbT* | | alkanal monooxygenase (FMN-linked) | -11.37 | 0.011 |
|  | *HFX_6205* | | hypothetical protein | -10.11 | 0.022 |
|  | *htr* | | heme-based aerotactic transducer HemAT | -9.74 | 0.027 |
| **HM5_M** | *HFX_6026* | | hypothetical protein | -9.89 | 0.025 |
| **HM6_M** | *HFX_6466* | | hypothetical protein | -12.74 | 0.005 |
| **HM7_M** | *apt3* | | adenine phosphoribosyltransferase | -11.22 | 0.012 |
|  | *HFX_1537* | | hypothetical protein | -10.99 | 0.014 |
|  | *HFX_2395* | | hypothetical protein | -10.78 | 0.015 |
|  | *HFX_2088* | | hypothetical protein | -10.75 | 0.016 |
|  | *lysE* | | LysE family L-lysine efflux protein | -10.51 | 0.018 |
| **HM8_M** | *HFX_1677* | | hypothetical protein | -13.68 | 0.002 |
|  | *HFX_0988* | | phosphoesterase-like protein | -11.97 | 0.008 |
|  | *HFX_1671* | | hypothetical protein | -11.91 | 0.008 |
|  | *HFX_2028* | | hypothetical protein | -11.74 | 0.009 |
|  | *HFX_1374* | | type I phosphodiesterase/nucleotide pyrophosphatase | -11.44 | 0.011 |
| **HM9_M** | *HFX_1142* | | hypothetical protein | -19.13 | 0 |
|  | *HFX_0592* | | hypothetical protein | -10.48 | 0.018 |
|  | *hcaA1* | | oxidoreductase | -8.74 | 0.042 |
| **HM10_M** | *HFX_0076* | | hypothetical protein | -10.07 | 0.023 |
|  | *rps8P* | | 30S ribosomal protein S8P | -8.3 | 0.05 |
| **HM11_M** | *HFX_0311* | | riboflavin biosynthetic operon protein,  transtriptional regulator / riboflavin kinase, archaea type | -13.21 | 0.003 |
|  | *uspA* | | stress response protein | -12.84 | 0.004 |
|  | *HFX_2597* | | aromatic-L-amino-acid decarboxylase | -11.69 | 0.009 |
|  | *HFX_0505* | | hypothetical protein | -10.47 | 0.018 |
|  | *smc* | | chromosome partition protein | -10.22 | 0.021 |
| **HM12_M** | *HFX_1575* | | hypothetical protein | -14.14 | 0.002 |
|  | *mutT* | | mutT/NUDIX family protein | -12.96 | 0.004 |
|  | *HFX_2074* | | hypothetical protein | -11.72 | 0.009 |
|  | *coxB4* | | cytochrome c oxidase subunit II | -8.62 | 0.044 |
| **HM13_M** | *HFX_0238* | | hypothetical protein | -20.8 | 0 |
|  | *HFX_2557* | | hypothetical protein | -19.18 | 0 |
|  | *uppS* | | undecaprenyl diphosphate synthase | -17.67 | 0 |
|  | *HFX_1366* | | hypothetical protein | -15.93 | 0 |
|  | *ushA* | | 5'-nucleotidase; 2',3'-cyclic-nucleotide 2'-phosphodiesterase;  UDP-sugar hydrolase | -15.81 | 0 |
| **HM14_M** | *hat2* | | acetyltransferase | -20.04 | 0 |
|  | *usp26* | | universal stress protein | -17.68 | 0 |
|  | *rpoB"* | | DNA-directed RNA polymerase subunit B'' | -17.11 | 0 |
|  | *HFX_1028* | | hypothetical protein | -15.43 | 0 |
|  | *gvpD* | | gas-vesicle operon protein gvpD | -13.14 | 0.003 |
| **HM15_M** | *trxA_1* | | thioredoxin | -11.45 | 0.011 |
|  | *pps2* | | phosphoenolpyruvate synthase / pyruvate, water dikinase | -10.39 | 0.019 |
|  | *HFX_0934* | | hypothetical protein | -9.29 | 0.033 |
| **HM16_M** | *HFX_1537* | | hypothetical protein | -15.44 | 0 |
|  | *pgk* | | phosphoglycerate kinase | -15.16 | 0.001 |
|  | *HFX_1505* | | hypothetical protein | -12.83 | 0.004 |
|  | *trkA* | | Trk potassium uptake system protein | -12.66 | 0.005 |
|  | *HFX_1489* | | hypothetical protein | -12.33 | 0.006 |
| **HM1_S** | *livG* | | ABC-type branched-chain amino acid transport systems,  ATP-binding protein I | -17.09 | 0 |
|  | *HFX_1497* | | hypothetical protein | -11.62 | 0.01 |
|  | *HFX_2088* | | hypothetical protein | -11.24 | 0.012 |
|  | *HFX_2739* | | ferredoxin | -9.86 | 0.025 |
|  | *nce1* | | Na+/Ca2+-exchanging protein | -9.12 | 0.035 |
| **HM2_S** | *HFX_0779* | | hypothetical protein | -14.27 | 0.001 |
|  | *HFX_3022* | | hypothetical protein | -12.32 | 0.006 |
|  | *tadC* | | type II secretion system, transmembrane protein TadC | -11.49 | 0.01 |
|  | *ilvE* | | branched-chain amino acid aminotransferase | -10.02 | 0.023 |
|  | *tp27* | | putative transport protein | -9.07 | 0.036 |
| **HM3_S** | *HFX_0324* | | hypothetical protein | -13.64 | 0.002 |
|  | *HFX_2025* | | hypothetical protein | -13.46 | 0.003 |
|  | *HFX_1455* | | phosphotransferase | -13.61 | 0.005 |
|  | *HFX_0285* | | hypothetical protein | -12.43 | 0.006 |
|  | *gar1* | | H/ACA RNA-protein complex component Gar1 | -12.37 | 0.006 |
| **HM4_S** | *HFX_0846* | | hypothetical protein | -12.65 | 0.005 |
|  | *HFX_1262* | | hypothetical protein | -11.68 | 0.009 |
|  | *HFX_1254* | | hypothetical protein | -11.26 | 0.012 |
|  | *HFX_2934* | | nonhistone chromosomal protein | -9.85 | 0.025 |
|  | *oppD2* | | oligopeptide ABC transporter ATPase component | -9.17 | 0.035 |
| **HM5_S** | *no targets* | | no targets |  |  |
| **HM6_S** | *xthA* | | DNA-(apurinic or apyrimidinic site) lyase / deoxyribonuclease IV | -12.43 | 0.006 |
|  | *dap2* | | dipeptidyl aminopeptidases/acylaminoacyl-peptidase | -11.69 | 0.009 |
|  | *HFX_0771* | | hypothetical protein | -11 | 0.014 |
|  | *HFX_0201* | | hypothetical protein | -10.68 | 0.016 |
|  | *yfmJ1* | | NADPH2:quinone reductase | -9.91 | 0.024 |
| **HM7_S** | *HFX_0627* | | hypothetical protein | -15.89 | 0 |
|  | *acnA* | | aconitate hydratase | -12.28 | 0.006 |
|  | *HFX_1005* | | hypothetical protein | -9.98 | 0.024 |
|  | *arsR (HFX_1218)* | | putative transcriptional regulator, ArsR family | -9.83 | 0.025 |
|  | *HFX_3006* | | hypothetical protein | -9.81 | 0.026 |
| **HM8_S** | *atpl* | | A-type ATP synthase subunit I | -11.65 | 0.009 |
|  | *HFX_0366* | | hypothetical protein | -10.91 | 0.014 |
|  | *gatD* | | glutamyl-tRNA(Gln) amidotransferase subunit D | -10.63 | 0.017 |
|  | *atpF* | | A-type ATP synthase subunit F | -9.51 | 0.03 |
|  | *gvpJ* | | gas-vesicle operon protein gvpJ | -8.47 | 0.047 |
| **HM1_A** | *HFX_2686* | | hypothetical protein | -18.72 | 0 |
|  | *ilvE* | | branched-chain amino acid aminotransferase | -11.09 | 0.013 |
|  | *secG* | | preprotein translocase subunit SecG | -9.81 | 0.026 |
|  | *dppB* | | dipeptide ABC transporter permease | -9.8 | 0.026 |
|  | *ecm4* | | glutathione S-transferase | -9.44 | 0.031 |
| **HM2_A** | *HFX_0013* | | ski2-like helicase | -11.08 | 0.013 |
|  | *HFX_2418* | | hypothetical protein | -11.01 | 0.014 |
|  | *menD* | | 2-oxoglutarate decarboxylase;  2-succinyl-6-hydroxy-2,4-cyclohexadiene-1-carboxylate synthase /  2-succinyl-5-enolpyruvyl-6-hydroxy-3-cyclohexene-1-carboxylate synthase | -9.48 | 0.03 |
|  | *gloB* | | metallo-beta-lactamase superfamily protein | -8.62 | 0.044 |
| **HM3_A** | *HFX_1910* | | hypothetical protein | -13.52 | 0.003 |
|  | *HFX_0630* | | hypothetical protein | -9.25 | 0.033 |
|  | *HFX_0770* | | hypothetical protein | -8.93 | 0.038 |
| **HM4_A** | *HFX-0853* | | hypothetical protein | -10.6 | 0.017 |
|  | *HFX_2853* | | hypothetical protein | -8.93 | 0.038 |
|  | *nosD1* | | copper-binding protein | -8.46 | 0.047 |
|  | *HFX_0589* | | hypothetical protein | -8.43 | 0.047 |
| **HM5_A** | *HFX_2594* | | hypothetical protein | -10.83 | 0.015 |
|  | *gtl3* | | putative glycosyltransferase, type 2 | -9.48 | 0.03 |
|  | *cysA* | | thiosulfate sulfurtransferase | -8.8 | 0.041 |
| **HM6_A** | *PPS2* | | phosphoenolpyruvate synthase / pyruvate, water dikinase | -14.57 | 0.001 |
|  | *HFX_2516* | | kynureninase | -12.5 | 0.005 |
|  | *citZ* | | citrate (si)-synthase | -12.24 | 0.006 |
|  | *secY* | | preprotein translocase subunit SecY | -11.94 | 0.008 |
|  | *HFX_1829* | mercuric transport protein | | -11.66 | 0.009 |
